# Harmonizing Vietnamese traditional and western medicine: a digital health blueprint and the IMKE architectural framework

**DOI:** 10.3389/fdgth.2026.1928965

**Published:** 2026-10-01

**Authors:** Anh Nguyen Thi Dieu, Hieu Le Ngoc

**Affiliations:** 1 Van Hien University, Ho Chi Minh City, Vietnam; 2 Posts and Telecommunications Institute of Technology, Ho Chi Minh City, Vietnam

**Keywords:** clinical decision support systems (CDSS), digital health, herb-drug interactions (HDIs), integrative medicine, medical informatics, pharmacovigilance, Vietnamese traditional medicine (VTM)

## Abstract

The concurrent use of traditional and Western medicine is a global healthcare reality. In countries like Vietnam, dual-paradigm clinical guidelines actively mandate integrated treatments for high-prevalence chronic diseases. However, safe integration at the point of care is severely hindered by data fragmentation, profound epistemological differences, and a critical “shadow pharmacovigilance” crisis regarding underreported herb-drug interactions (HDIs). This paper analyzes the state-of-the-art of Artificial Intelligence (AI) in integrative healthcare and proposes the Integrative Medicine Knowledge Engine (IMKE), a novel architectural blueprint designed to safely bridge Vietnamese traditional medicine (VTM) and Western biomedicine. Shifting from siloed technical models to a clinical-first perspective, the IMKE conceptualizes a three-stage digital health pipeline: (1) advanced medical text mining to digitize unstructured traditional practitioner notes; (2) an Integrative Knowledge Graph (IKG) that maps holistic VTM diagnostic patterns to Western pharmacological databases and ICD-10 standards; and (3) an AI-driven Clinical Decision Support System (CDSS) for proactive HDI safety monitoring. We illustrate this architectural data flow through three clinical scenarios—Essential Hypertension, Type 2 Diabetes, and Rheumatoid Arthritis. These scenarios demonstrate the system’s theoretical capacity to retrieve culturally appropriate treatments, identify cross-paradigm therapeutic synergies, and generate explainable, mechanistic safety alerts that augment physician judgment while mitigating clinical alert fatigue. Ultimately, the IMKE framework provides a scalable, culturally inclusive roadmap for digital health. By translating centuries of experiential medical wisdom into an interoperable, computationally safeguarded infrastructure, this blueprint sets the foundation for evidence-based integrative care, future retrospective clinical validation, and enhanced patient safety.

## 1 Introduction

Healthcare systems globally face an escalating burden of chronic non-communicable diseases (NCDs), which account for nearly 75% of all mortality [1]. While Western biomedicine has achieved extraordinary clinical success in acute trauma, surgical intervention, and infectious diseases through targeted pharmacological protocols [3], there is growing recognition that this reductionist approach struggles to fully address the complex, systemic nature of chronic morbidities [2]. By isolating individual biological targets, modern biomedicine often overlooks the underlying physiological dysregulations driving conditions like metabolic syndrome, autoimmune disorders, and neurodegeneration [4, 5].

Consequently, patients increasingly turn to Eastern medical traditions, such as traditional Chinese medicine (TCM) and its deeply localized derivative, Vietnamese traditional medicine (VTM). These holistic systems emphasize the harmonization of the human body through personalized, multi-compound herbal formulas targeting functional “patterns of disharmony” rather than isolated molecular biomarkers [6, 7]. The World Health Organization (WHO) actively advocates for the integration of traditional medicine into national healthcare systems, recognizing its role in evidence-based complementary care for billions globally [8]. In Vietnam, this integration is a highly active clinical reality. VTM is practiced alongside Western biomedicine in over 90% of provincial hospitals, supported by national policies officially mandating dual-paradigm treatments for high-prevalence chronic diseases [9, 10].

Despite this widespread clinical co-utilization, integrative care at the point of delivery remains dangerously fragmented, creating severe barriers to patient safety. This fragmentation is driven by three foundational gaps. First, a knowledge gap: VTM therapeutic wisdom remains trapped in unstructured textual formats—ranging from ancient classical manuscripts to modern, handwritten practitioner notes—rendering it inaccessible for digital health integration and modern clinical workflows [11, 12]. Second, an evidence gap: a profound epistemological incompatibility exists between Western medicine’s demand for molecular biomarkers and VTM’s phenomenological diagnoses, hindering health data interoperability [13, 14]. Third, and most critically, a safety gap: the underreported risk of herb-drug interactions (HDIs). Epidemiological surveys reveal that over 42% of patients concurrently using pharmaceutical drugs and traditional herbal remedies do not disclose this co-administration to their primary physicians. This creates a massive “shadow pharmacovigilance” crisis, leaving clinicians prescribing in the dark [15, 16].

Digital health technologies, particularly Artificial Intelligence (AI), offer a viable bridge for these epistemological and structural divides. The convergence of large language models (LLMs) [17], biomedical knowledge graphs (KGs) [18, 19], and graph neural networks (GNNs) [20] now provides the computational infrastructure necessary to unify divergent medical data. Predictive analytics driven by deep learning on electronic health records [21] and big data machine learning [22] have already catalyzed a fundamental shift in evidence-based medicine. Furthermore, recent dual-paradigm clinical guidelines have demonstrated the clinical viability of integrated protocols [23]. Recently, significant methodological advancements have been proposed to organically integrate TCM and Western medicine clinical practice guidelines [84, 85]. However, while prior research has successfully applied AI to isolated traditional medicine tasks—such as TCM symptom mapping or molecular docking for active compounds [24, 25]—these efforts remain siloed. Existing platforms function primarily as static pharmacological databases for researchers rather than dynamic clinical tools, critically lacking the capacity for real-time safety inference and explainable clinical decision support (CDSS) at the point of care [26, 27].

Figure 1 illustrates the conceptual role of the IMKE as a bridge between the VTM and Western biomedical paradigms.

**Figure 1:**
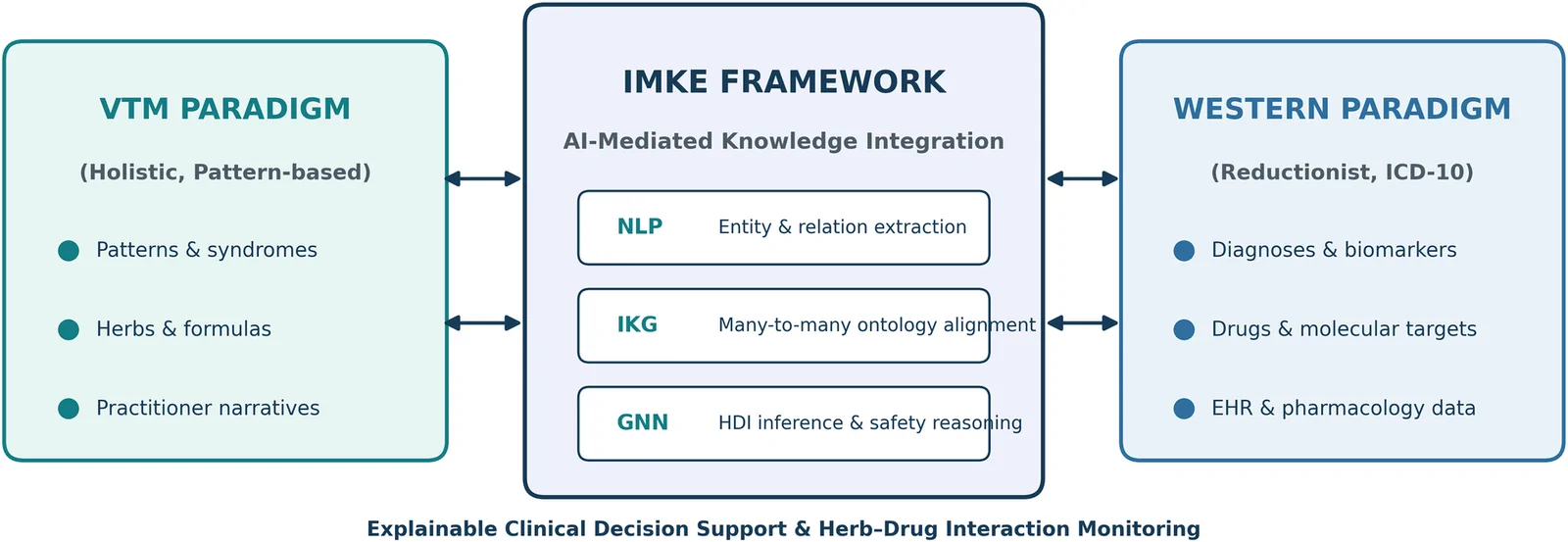
Block-diagram representation of the IMKE framework as an AI-mediated computational bridge between the holistic, pattern-based VTM paradigm and reductionist, ICD-10-oriented Western biomedicine.

To address these urgent clinical bottlenecks, this paper presents a comprehensive review of AI in integrative healthcare and proposes the Integrative Medicine Knowledge Engine (IMKE). Rather than presenting an isolated algorithm, this paper introduces a comprehensive digital health blueprint designed to safely harmonize VTM and Western medicine in clinical practice. The contributions of this work are threefold:

State-of-the-art analysis: We critically evaluate existing traditional medicine AI, identifying the critical absence of end-to-end, cross-paradigm safety monitoring systems.

Architectural blueprint: We conceptualize the IMKE framework, a three-stage clinical informatics pipeline comprising an advanced Natural Language Processing (NLP) module for unstructured VTM knowledge extraction, an integrative knowledge graph (IKG) for cross-paradigm ontology alignment, and an AI-driven module for predictive HDI safety monitoring.

Simulated operational scenarios: We illustrate the architecture’s logical data flow and reasoning capacity through three simulated clinical use-cases. These demonstrate how the IMKE translates complex data into explainable, actionable CDSS alerts that augment physician judgment and mitigate clinical alert fatigue.

The central hypothesis of this work is that fusing VTM phenomenological concepts with Western pharmacological data into a multi-relational knowledge representation, combined with relation-aware computational reasoning, can improve the sensitivity and interpretability of novel HDI detection compared with single-paradigm databases.

By formalizing this architecture, this work provides a scalable, mathematically rigorous, and culturally sensitive template for AI-mediated integrative healthcare, offering a robust foundation for patient safety and future empirical deployment in Vietnam and globally.

The remainder of this paper is structured as follows. Section 2 presents the state-of-the-art in integrative medicine AI. Section 3 details the clinical and technical design rationale of the IMKE architecture. Section 4 illustrates the framework through simulated clinical scenarios. Section 5 discusses open clinical challenges, regulatory perspectives, and the future development roadmap. Section 6 concludes the paper.

## 2 State-of-the-art in integrative medicine AI

To establish a rigorous clinical informatics and architectural foundation for the IMKE framework, this section comprehensively reviews the current landscape of artificial intelligence (AI) applied to traditional Asian medicine and Western integrative practices. Guided by clinical insights and expert consultations with practitioners from the Hospital of Traditional Medicine,^1^ Ho Chi Minh City, this review critically evaluates existing computational frameworks across divergent medical paradigms to identify pivotal gaps in care delivery and patient safety.

### 2.1 Search strategy and divergent paradigms

A comprehensive literature search was conducted across IEEE Xplore, PubMed, and Scopus, including articles published from January 2014 to May 2024, restricted to English and Vietnamese languages. The precise search string utilized combinations of terms related to artificial intelligence, traditional medicine, integrative medicine, knowledge graphs, clinical decision support, and herb–drug interactions. The fundamental clinical challenge identified across the literature is the epistemological divide between Western and Eastern medicine.

Western medicine’s epistemology is rooted in Cartesian reductionism, decomposing biological complexity into measurable components (genes, proteins) targeted with precise interventions [28]. While this paradigm has generated transformative clinical advances validated by randomized controlled trials (RCTs) [29], it struggles with chronic, multifactorial diseases where causality is distributed across systemic biological networks [4]. Conversely, Vietnamese traditional medicine (VTM)—systematized in the 18th-century text Hải Thượng Y Tông Tâm Lĩnh—operates on a holistic framework [6]. Diagnosis is pattern-based (e.g., “liver Yang rising”), integrating phenomenological descriptors that resist quantification without substantial ontological translation [5]. As Monnais (2019) observes, this creates a fundamental language barrier that persists in contemporary clinical workflows and regulatory frameworks, hindering collaborative care [14].

### 2.2 Taxonomy of AI applications in traditional medicine

The application of AI to traditional medicine has evolved from rudimentary digitization to deep-learning-driven multi-domain reasoning [11]. To provide a comprehensive technical overview, Table 1 summarizes the primary machine learning architectures currently applied in integrative medicine, highlighting their representative studies and inherent limitations.

**Table 1:** Artificial intelligence and machine learning techniques in integrative medicine research.

Technique	Primary role in integrative medicine	Representative application	Key limitation
Transformer-based NLP (BERT, PhoBERT)	Extraction of entities and semantic relations from traditional Chinese medicine (TCM) and Vietnamese traditional medicine (VTM) literature	Clinical concept recognition, herb–disease relation extraction, terminology normalization	Limited performance on historical texts and classical Sino-Vietnamese terminology
Graph neural networks (GNNs)	Modeling complex interactions among herbs, compounds, genes, diseases, and pharmaceuticals	Herb–drug interaction prediction, link prediction, multi-hop reasoning in knowledge graphs	Cold-start issues and dependence on large, well-annotated graph datasets
Knowledge graph embedding (TransE, RotatE)	Learning latent representations of biomedical and traditional medicine entities and relations	Knowledge graph completion, relation inference, similarity-based discovery	Difficulty generalizing to unseen entities and emerging concepts
Recurrent neural networks (LSTM)	Temporal analysis of longitudinal clinical and electronic health record (EHR) data	Disease progression modeling and treatment outcome prediction	Susceptible to long-range dependency and gradient vanishing problems
Network pharmacology	Systems-level exploration of multi-component and multi-target therapeutic mechanisms	Identification of molecular targets and pathway enrichment analysis for herbal formulas	Reliance on incomplete or heterogeneous biological interaction databases
Explainable AI (SHAP, LIME)	Enhancing transparency and interpretability of clinical decision support systems (CDSS)	Explanation of risk predictions, treatment recommendations, and interaction alerts	Post-hoc explanations may not fully reflect underlying model behavior

The application of AI to traditional medicine has evolved from rudimentary digitization to deep-learning-driven multi-domain reasoning [30]. Based on our comprehensive review, we categorize the current research landscape into three primary technological pillars (Figure 2):

**Figure 2:**
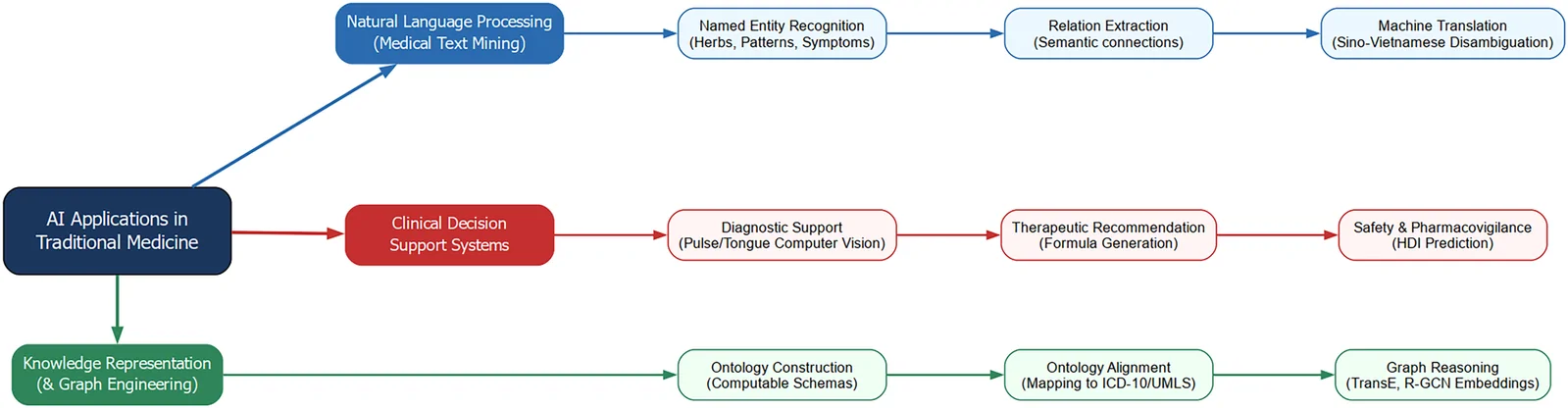
Taxonomy of artificial intelligence applications in traditional medicine, categorized into three core technological pillars: NLP, knowledge representation, and CDSS.

Natural language processing (NLP) for medical text mining: The foundational step involves extracting traditional concepts (herbs, formulas, symptoms) from unstructured texts, a task fundamentally challenged by the complexity of biomedical entity and adverse effect recognition [31]. Transformer architectures [32] have revolutionized this domain. Domain-specific pre-training—demonstrated by models such as BioBERT [33], ClinicalBERT [34], and SciBERT [35]—has substantially improved performance on biomedical NLP tasks. However, while these models excel in English, VTM presents a unique challenge spanning three linguistic registers—modern Vietnamese, Sino-Vietnamese, and classical Han-Nom. Although PhoBERT provides a robust foundation for Vietnamese NLP [12], domain-specific relation extraction for VTM remains an unsolved challenge.

Knowledge representation and graph engineering (KG): KGs are heterogeneous networks representing real-world entities and typed semantic relationships [36]. In biomedicine, KGs like the UMLS metathesaurus and SPOKE have become central architectural patterns for reasoning over clinical knowledge [18]. For traditional medicine, the core challenge lies in ontology alignment—mapping pattern-based diagnoses to Western biomedical standards (e.g., ICD-10) and utilizing graph embedding techniques (e.g., TransE, R-GCN) to preserve semantic structures [37, 38]. Recent comprehensive reviews [39] and methodological advances [40] in TCM knowledge graph construction underscore the immense clinical value of these technologies, yet domain-specific ontology alignment for VTM remains largely unsolved.

Clinical Decision Support Systems (CDSS): The ultimate translational goal of AI is to augment human clinical judgment [27]. While AI has been applied to diagnostic instruments like tongue and pulse analysis [41], there is a critical lack of end-to-end CDSS frameworks that monitor cross-paradigm safety, specifically herb-drug interactions (HDIs).

Based on our literature review, we categorize the current research landscape into three primary technological pillars (Figure 2):

### 2.3 State-of-the-art frameworks: a comparative analysis

Over the past decade, significant efforts have been made to digitize traditional Chinese medicine (TCM) and explore network pharmacology [30]—a paradigm first established by Hopkins to transition drug discovery from reductionist single-target models to systems-level, multi-target analyses [42]. Landmark databases such as TCMID [43], SymMap [25], and HERB [24] have provided extensive mappings between herbs, chemical compounds, and biological targets. Beyond static databases, recent conceptual advances have moved toward artificial-intelligence-based precision TCM using network pharmacology [86] and complex-systems evidence frameworks [87]. However, as noted by clinical experts, these platforms and conceptual frameworks do not yet constitute dynamic cross-paradigm clinical safety tools.

Table 2 compares these databases, evidence frameworks, and guideline methodologies with the proposed IMKE architecture.

**Table 2:** State-of-the-Art comparison of integrative medicine databases, evidence frameworks, guideline methodologies, and the proposed IMKE architecture.

Framework/system	Traditional system focus	Unstructured NLP pipeline supported?	VTM/TCM to Western ontology mapping	Cross-paradigm Safety (HDI Prediction)	Real-time CDSS UI/clinical alerts	Access type
TCMID [43]	TCM	No (Manual Curation)	Basic (Disease level)	No (Database lookup only)	No	Open Access DB
SymMap [25]	TCM	No (Manual Curation)	Yes (Symptom & Target level)	No	No	Open Access DB
HERB [24]	TCM	No	Yes (Gene/Protein level)	No	No	Open Access DB
SPOKE [18]	Western	Yes (via external tools)	N/A (Western only)	Yes (Drug-Drug interactions)	Partially (Research)	Academic
Wei et al. (2026) [87]	TCM	No	No	No (Focuses on evidence hierarchy)	No	Academic
Zhang et al. (2024) [86]	TCM	No	Yes (Targets)	No (Focuses on precision efficacy)	No	Academic
Jin et al. (2023) [84]	TCM & Western	No	Conceptual (Guideline level)	No (Guideline methodology)	No	Academic
Xie et al. (2024) [85]	TCM & Western	No	Conceptual (Guideline level)	No (Guideline implementation)	No	Academic
IMKE (proposed)	VTM & Western	Yes (Context-aware Vietnamese NLP; candidate encoders are illustrative)	Yes (Many-to-many mapping to ICD-10 & UMLS)	Yes (Relation-aware multi-hop HDI inference)	Yes (Explainable clinical alert concept)	Architectural blueprint

The four recent conceptual contributions clarify the specific research gap addressed by IMKE. Zhang et al. position network pharmacology as a foundation for AI-enabled precision TCM by linking herbal components to molecular targets and efficacy-oriented predictions [86]. Wei et al. advance this systems perspective at the evidence level through a four-phase framework intended to organize complex, multi-source Chinese medicine evidence [87]. These contributions strengthen the theoretical basis for systems-oriented traditional medicine research, but neither specifies an end-to-end clinical informatics pipeline for extracting unstructured VTM records, aligning them with Western clinical terminologies, and generating patient-level HDI safety alerts.

Jin et al. and Xie et al. address a complementary implementation layer by proposing methodological principles for the organic integration of traditional Chinese and Western medicine clinical practice guidelines [84, 85]. Their work provides an important governance and guideline-development foundation, whereas IMKE focuses on the computational operationalization of integrative guidance at the point of care. Accordingly, IMKE’s proposed novelty lies not in replacing these evidence and guideline frameworks, but in connecting them through a technology-agnostic architecture that combines multilingual NLP, many-to-many ontology mapping, multi-relational HDI inference, and explainable CDSS delivery.

### 2.4 Herb-drug interaction safety: the critical clinical gap

Herb-drug interactions (HDIs) represent the most consequential challenge at the interface of integrative medicine. HDIs encompass both pharmacokinetic interactions (e.g., CYP450 enzyme modulation) and pharmacodynamic interactions (synergistic or antagonistic effects) [44]. Epidemiological studies indicate that clinically significant HDIs affect 5%–10% of patients using concurrent therapies, with a vast majority of cases undetected by routine pharmacovigilance [16].

Foundational data-driven approaches have long demonstrated the power of predicting unknown drug effects and interactions from large-scale clinical databases [45]. Building on this, while recent advances have utilized graph neural networks (GNNs) to predict drug-drug interactions (DDIs) with high accuracy [20, 46, 47], and data mining techniques have successfully extracted interactions from clinical text sources [48], HDI prediction specifically tailored to VTM remains sparse. Although machine learning classifiers have shown promise in predicting TCM herb-drug pairs [49], the complexity of herbal formulations—where multiple active compounds simultaneously modulate different pathways—renders simple rule-based detection inadequate, necessitating multi-hop graph reasoning capabilities [37].

Recent studies published in IEEE Access have demonstrated the immense potential of graph neural networks in modern healthcare [50]. Specifically, state-of-the-art architectures have successfully integrated Transformer models with biomedical knowledge graphs (KITE-DDI) [46] and utilized Graph Attention Networks (KGAT) [47] to accurately predict complex drug-drug interactions, providing a strong methodological baseline for the IMKE’s safety module. The complexity of herbal formulations—where multiple active compounds simultaneously modulate different pathways—renders simple rule-based detection inadequate, necessitating multi-hop graph reasoning capabilities [37]. To illustrate the clinical severity of this gap, Table 3 details key examples of highly consequential herb-drug interactions that the IMKE framework is designed to detect and prevent.

**Table 3:** Representative herb–drug interactions requiring GNN-Based detection in the Integrative Medicine Knowledge Engine (IMKE).

Herbal medicine (Vietnamese/scientific name)	Major bioactive compound(s)	Conventional drug	Interaction mechanism	Clinical relevance
Câu đằng (Uncaria rhynchophylla)	Rhynchophylline	Lisinopril	Additive antihypertensive effect	High risk of excessive blood pressure reduction; close monitoring recommended
Dây khổ qua (Momordica charantia)	Charantin, Polypeptide-p	Glibenclamide	Synergistic glucose-lowering effect	Increased risk of severe hypoglycaemia; dosage adjustment may be required
Ma hoàng (Ephedra sinica)	Ephedrine	Monoamine Oxidase Inhibitors/β-blockers	Pharmacodynamic antagonism and sympathomimetic enhancement	Potential hypertensive crisis; generally contraindicated
Cam thảo (Glycyrrhiza uralensis)	Glycyrrhizin	Corticosteroids/Digoxin	Potentiation of hypokalaemic effects	Elevated risk of electrolyte imbalance and cardiac toxicity; serum potassium monitoring required
Đand sâm (Salvia miltiorrhiza)	Tanshinone IIA, aalvianolic acid	Warfarin	Enhancement of anticoagulant activity	Increased bleeding risk; frequent INR monitoring recommended

### 2.5 Policy, cultural dimensions, and institutional alignment

The integration of traditional and Western medicine is highly dependent on institutional and regulatory frameworks. Vietnam’s Ministry of Health (MoH) actively promotes dual-paradigm clinical protocols. A landmark expression of this policy is the official MoH Guidelines (Decision No. 3890/QD-BYT, 2020; updated 2026), which establish standardized protocols combining VTM pattern-based diagnosis with ICD-10-aligned Western differential diagnosis for high-prevalence conditions [9, 10].

Crucially, these guidelines mandate that VTM practitioners must not rely solely on traditional medicine but must combine it with Western diagnostic methods to ensure patient safety. This regulatory foundation provides the IMKE with an authoritative, operationally tested schema for knowledge graph construction. However, sociological reviews confirm that even well-designed policies often produce institutional coexistence rather than genuine collaboration [13, 51]. Discussions with practitioners highlight that without computational mediation, the risk of undetected HDIs remains high.

### 2.6 Identification of research gaps and IMKE’s positioning

Synthesizing the literature review and comparative analysis (Table 1), we identify three pivotal gaps that the IMKE framework is designed to bridge:

The end-to-end processing gap: Existing databases (TCMID, SymMap) lack automated, VTM-specific NLP pipelines capable of dynamically extracting multi-relational knowledge from unstructured, multi-script Vietnamese texts.

The cross-paradigm safety gap: Current platforms utilize the principles of network pharmacology [42] primarily to prove therapeutic efficacy (e.g., identifying target overlap for disease treatment), but largely ignore predictive safety. No existing traditional medicine platform features automated, GNN-driven prediction of novel herb-drug interactions spanning both VTM and Western pharmacopeia.

The clinical translation gap: Despite the existence of MoH integrative guidelines [9], no computational system has operationalized these rules into a machine-readable format integrated with an explainable CDSS.

The IMKE addresses these gaps by treating the MoH guidelines as the authoritative seed for its integrative knowledge graph (IKG), grounding all mappings in regulatory-grade evidence before extending into probabilistic GNN-based HDI discovery. The architectural blueprint to achieve this is detailed in Section 3.

## 3 Methodology: the IMKE framework

As a Hypothesis and Theory article, the IMKE defines functional requirements and testable methodological pathways rather than prescribing a fixed implementation stack. Technologies named below—including PhoBERT, Neo4j, R-GCN, SHAP, Docker, and particular optimization functions—are illustrative candidate instantiations selected to demonstrate how the conceptual requirements might be operationalized. The equations, query examples, interface mockups, and evaluation dimensions are therefore explanatory artifacts rather than a finalized protocol, trained system, or claim of achieved performance. Future empirical studies must compare alternative encoders, graph stores, relation-aware learning methods, explanation techniques, and deployment architectures before any implementation choice is fixed.

The IMKE framework is designed as a modular, three-stage architectural pipeline that transforms unstructured Vietnamese traditional medicine (VTM) knowledge into actionable, computable clinical guidance. The architecture is conceptualized to operate in a real-time clinical environment, processing multi-modal patient data—combining Western electronic health records (EHR) and VTM practitioner notes—through successive layers of NLP processing, knowledge graph reasoning, and graph-neural-network-based safety prediction.

Figure 3 summarizes the proposed functional architecture and its three interconnected layers.

**Figure 3:**
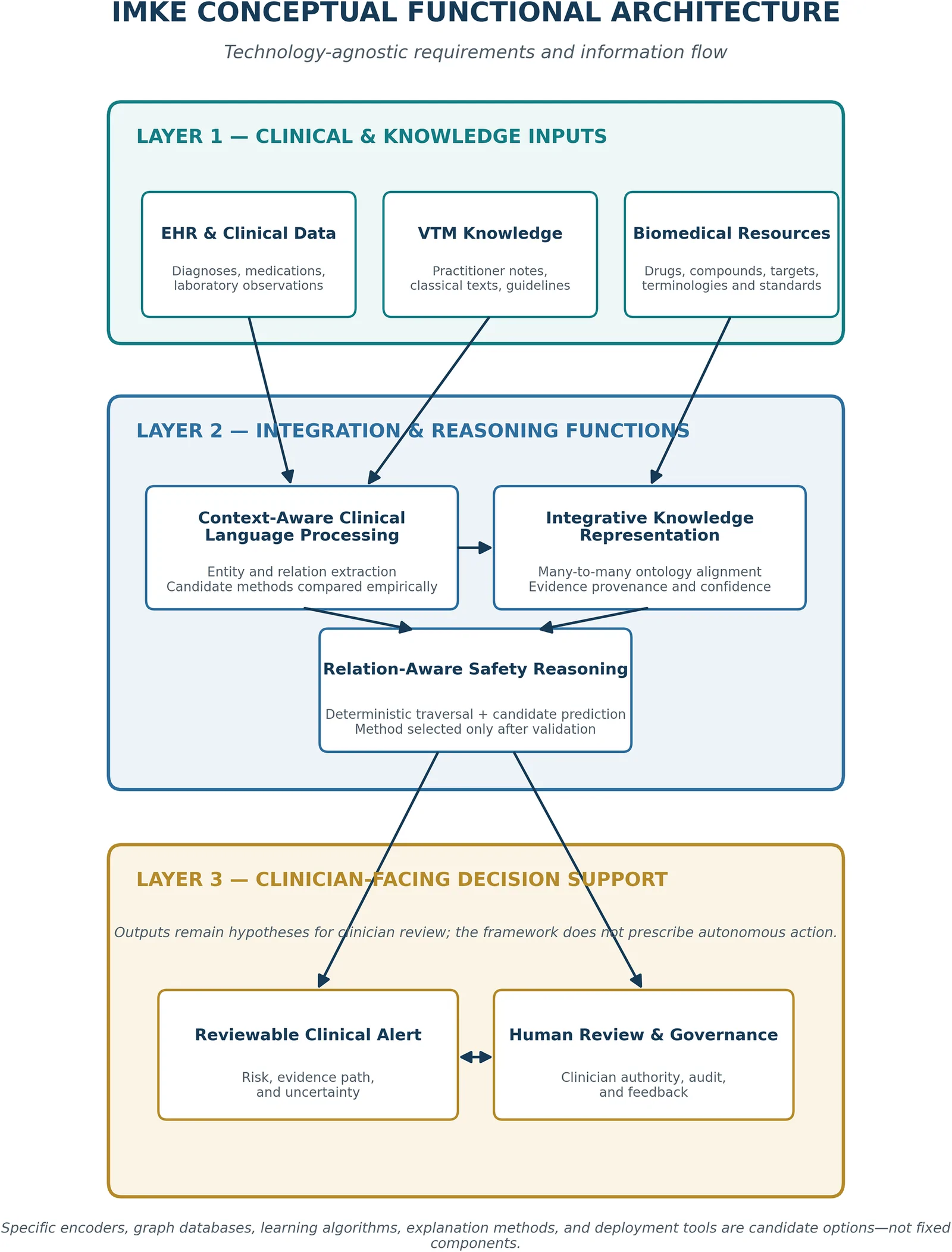
Technology-agnostic functional architecture of the IMKE. The three layers specify clinical and knowledge inputs, integration and relation-aware reasoning requirements, and clinician-facing decision support with human governance. Named software and algorithms are not prescribed components.

### 3.1 Stage 1: NLP-based knowledge extraction and design rationale

The foundation of the IMKE is a specialized NLP pipeline that transforms unstructured VTM texts into machine-readable structured triples: (Entity_1, Relationship, Entity_2). This pipeline addresses the unique linguistic complexity of the VTM corpus, which spans three registers: modern Vietnamese, Sino-Vietnamese, and classical Han-Nom scripts.

#### 3.1.1 Data sources and corpus construction

The framework’s primary corpus leverages regulatory-grade documents to ensure clinical reliability. The core structured knowledge source is the Vietnamese Ministry of Health’s (MoH) official integrative medicine guidelines (2020) [9] and its 2026 expansion [10]. These protocols provide institutionally validated dual-paradigm diagnostic criteria, bat cuong (eight-principle) classifications, classical herbal formulae, and ICD-10-aligned Western guidance. This authoritative seed is supplemented by canonical VTM classical texts [7, 52], contemporary peer-reviewed pharmacological studies [16], and the Vietnamese National Formulary.

#### 3.1.2 Named entity recognition (NER): context-aware Vietnamese encoders

The NER module identifies five entity classes: Herbs, Symptoms, VTM Patterns of Disharmony, Western Diseases, and Therapeutic Actions.

Architectural considerations: The encoder backbone of the NLP pipeline necessitates an advanced, context-aware Transformer architecture to handle linguistic complexity. For instance, localized foundation models such as PhoBERT-base [12] serve as illustrative options alongside general multilingual models such as mBERT. Because Vietnamese occupies only a subset of multilingual training corpora, generic tokenization may increase out-of-vocabulary fragmentation, whereas models using syllable-aware byte-pair encoding are theoretically better aligned with Vietnamese linguistic structure. Consequently, fine-tuning a domain-specific contextual encoder—for example, following principles demonstrated by BioBERT [33]—is hypothesized to improve the representation of semantic boundaries and enable attention over clinically meaningful concepts rather than fragmented syllables. The optimal encoder and tokenization strategy remain empirical questions to be evaluated against multilingual, language-specific, and domain-adapted alternatives.

#### 3.1.3 Relation extraction and triple generation

Following NER, a joint entity-relation extraction architecture [53] classifies entity pairs into predefined relation types derived from VTM theory and Western pharmacology. Table 4 outlines the proposed ontology relation labels extracted by the NLP module to construct the IKG.

**Table 4:** Proposed relation extraction labels for the IMKE ontology.

Category	Relation label	Description/example
Therapeutic	TREATS	Herb/Formula treats a VTM Pattern or Disease
Compositional	CONTAINS	Herb contains an Active Compound
Pharmacodynamic	SYNERGIZES_WITH	Additive or synergistic therapeutic effect
Pharmacokinetic	INHIBITS	Compound inhibits a metabolic enzyme (e.g., CYP450)
Toxicological	CAUSES_ADVERSE_EVENT	Interaction directly leads to clinical toxicity
Contraindication	CONTRAINDICATED_WITH	Strictly prohibited combination

For example, from unstructured text, the system extracts: (*Glycyrrhiza uralensis*)—[HARMONIZES]→ (Toxic_Compounds). These structured triples form the raw input for the next stage.

### 3.2 Stage 2: integrative knowledge graph (IKG) construction

The IKG is the central data structure of the IMKE, formally defined as a directed, labeled multigraph G=(V,E,R,ψ), where V is the set of entity nodes, E⊆V×R×V is the set of typed relational edges, R is the set of relation types spanning both paradigms, and ψ:E→[0,1] is a confidence weight [36].

#### 3.2.1 Ontology schema design and data fusion

To enable cross-paradigm reasoning, the IKG enforces a strict ontology schema connecting macroscopic VTM phenomena to microscopic Western biomedicine:

VTM Sub-graph: (VTM_Pattern) [TREATS]- (Herbal_Formula) -[INCLUDES] (Herb)

Bridge Layer: (Herb) -[CONTAINS] (Active_Compound)

Western Sub-graph: (Active_Compound) -[TARGETS] (Gene/Protein) [INHIBITS/ACTIVATES]—(Western_Drug)—[TREATS] (Western_Disease)

Ontology alignment is performed via lexical matching, semantic embedding similarity, and expert manual curation [37]—a necessary multi-strategy approach given the global need for a standardized nomenclature bridging East and West [54]. The IKG fuses VTM triples with established external databases, including DrugBank [55], Gene Ontology, KEGG, and the UMLS Metathesaurus, utilizing an entity resolution algorithm to collapse synonymous nodes.

It is crucial to note that VTM patterns and Western ICD-10 diagnoses do not exhibit a direct one-to-one equivalence. The IKG is specifically architected to represent these relationships as many-to-many, evidence-qualified mappings, reflecting the complex clinical reality where a single Western disease may manifest as multiple VTM patterns, and vice versa.

Figure 4 illustrates the ontology schema and the proposed cross-paradigm alignment of traditional and biomedical entities.

**Figure 4:**
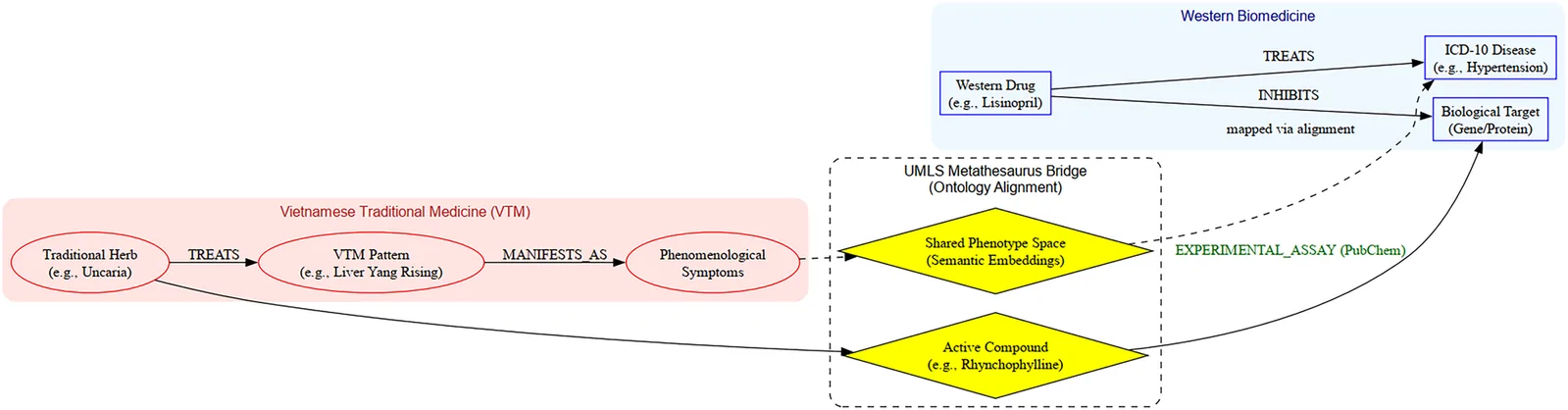
Ontology schema and cross-paradigm alignment. The diagram illustrates the mapping of Vietnamese Traditional Medicine entities (Patterns, Symptoms, Herbs) to Western biomedical entities (Diseases, Targets, Drugs) via the UMLS Metathesaurus bridge.

#### 3.2.2 Graph traversal and illustrative query pathway

When instantiated within a graph database management system (e.g., Neo4j), the IKG enables explicit topological reasoning. To identify whether a prescribed herb shares a biological target pathway with a pharmaceutical drug, a future CDSS could execute targeted graph traversals. For instance, to detect potential interactions between Uncaria rhynchophylla (Câu đằng) and Lisinopril, an illustrative Cypher Query Language (CQL) implementation could be structured as follows:

// IMKE Cypher Query for Cross-Paradigm Interaction Detection

MATCH path = (h:Herb {name: "Uncaria rhynchophylla"})

-[r1:CONTAINS]->(c:Compound)

-[r2:TARGETS]->(t:Target)

<-[r3:INHIBITS]-(d:WesternDrug {name: "Lisinopril"})

RETURN c.name AS Active_Ingredient, t.name AS Shared_Target, type(r2) AS Herb_Action, type(r3) AS Drug_Action

This query illustrates how multi-hop traversal could identify candidate mechanistic overlaps not represented as explicit pre-recorded interaction pairs. Equivalent traversals could be implemented using other graph query languages or storage systems.

### 3.3 Stage 3: clinical decision support system (CDSS)

The CDSS represents the proposed clinical interface to the IKG [27]. Its core functional requirement is to surface candidate Herb-Drug Interactions (HDIs) not captured by deterministic graph traversal alone, regardless of the specific predictive algorithm eventually selected.

#### 3.3.1 Relation-aware interaction prediction

Because biological interactions are inherently probabilistic, the IMKE framework proposes relation-aware graph learning as one methodological pathway for predictive safety monitoring. Architectures capable of handling heterogeneous edge types—such as Relational Graph Convolutional Networks (R-GCN) [38]—are illustrative candidates alongside standard GCNs [56], attention-based models [57], and inductive frameworks such as GraphSAGE [58]. Methods that treat all edges equivalently may fail to preserve the clinically opposite meanings of relations such as [TREATS] and [CAUSES_ADVERSE_EVENT]. An R-GCN-like architecture addresses this requirement by allocating relation-specific transformations to different edge types. One possible mathematical representation of this updating process is:

$$h_{i}^{\left(l+1\right)}=\sigma \left(\sum_{r\in R}\sum_{\;j\in N_{i}^{r}}\frac{1}{c_{i,r}}W_{r}^{\left(l\right)}h_{j}^{\left(l\right)}+W_{0}^{\left(l\right)}h_{i}^{\left(l\right)}\right)$$ (1)

where $h_{i}^{\left(l+1\right)}$ is the hidden representation of node i at layer l+1, R denotes the set of relation types, $N_{i}^{r}$ represents the set of neighbor indices of node i under relation r, $W_{r}^{\left(l\right)}$ is a relation-specific weight matrix, $c_{i,r}$ is a problem-specific normalization constant (e.g., computed based on node degrees), $W_{0}^{\left(l\right)}$ represents the self-loop weight matrix preserving the node’s original features, and σ is a non-linear activation function.

Equation 1 illustrates how relation-specific neighbor information and a self-loop contribution can be combined to update a node representation. Figure 5 illustrates relation-aware message passing using R-GCN as one possible model, rather than a required implementation.

**Figure 5:**
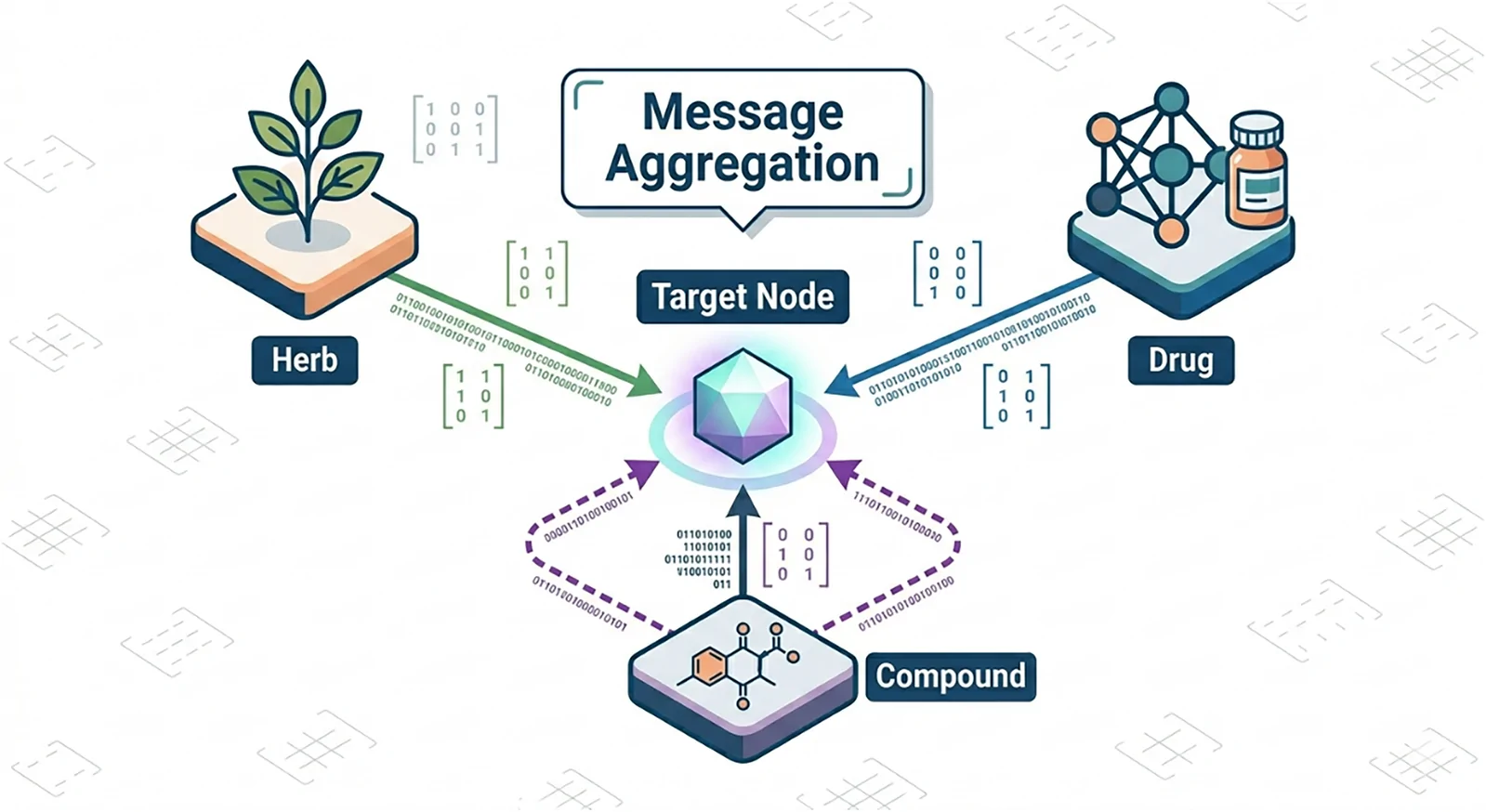
Illustrative R-GCN candidate showing a possible relation-aware message-passing mechanism for multi-relational Herb-Drug Interaction prediction. Other heterogeneous graph-learning architectures remain eligible for empirical comparison.

#### 3.3.2 Illustrative objective function for HDI prediction

The empirical training of a future predictive module would require optimizing an objective function against known HDI databases [16]. One standard option for link prediction is Binary Cross-Entropy Loss with Negative Sampling, although ranking, contrastive, and calibrated probabilistic objectives should also be compared. Under this illustrative option, the probability of an interaction edge can be modeled as: $e=(v_{\text{herb}},\text{INTERACTS\textbackslash{}\_WITH},v_{\text{drug}})$

$$L=-\sum_{(s,r,o)\in \Omega^{+}}\log\;\sigma (f(s,r,o))-\sum_{(s,r,o^{\prime })\in \Omega^{-}}\log\;(1-\sigma (f(s,r,o^{\prime })))$$ (2)

where $\Omega^{+}$ is the set of true interactions and $\Omega^{-}$ represents negatively sampled non-interacting pairs.

Equation 2 illustrates a possible training objective combining positive interaction examples and negatively sampled pairs; it is not a prescribed component of the conceptual framework. Figure 6 presents an illustrative CDSS dashboard showing how candidate interaction alerts and their supporting explanations could be communicated to clinicians; it does not display empirical model outputs.

**Figure 6:**
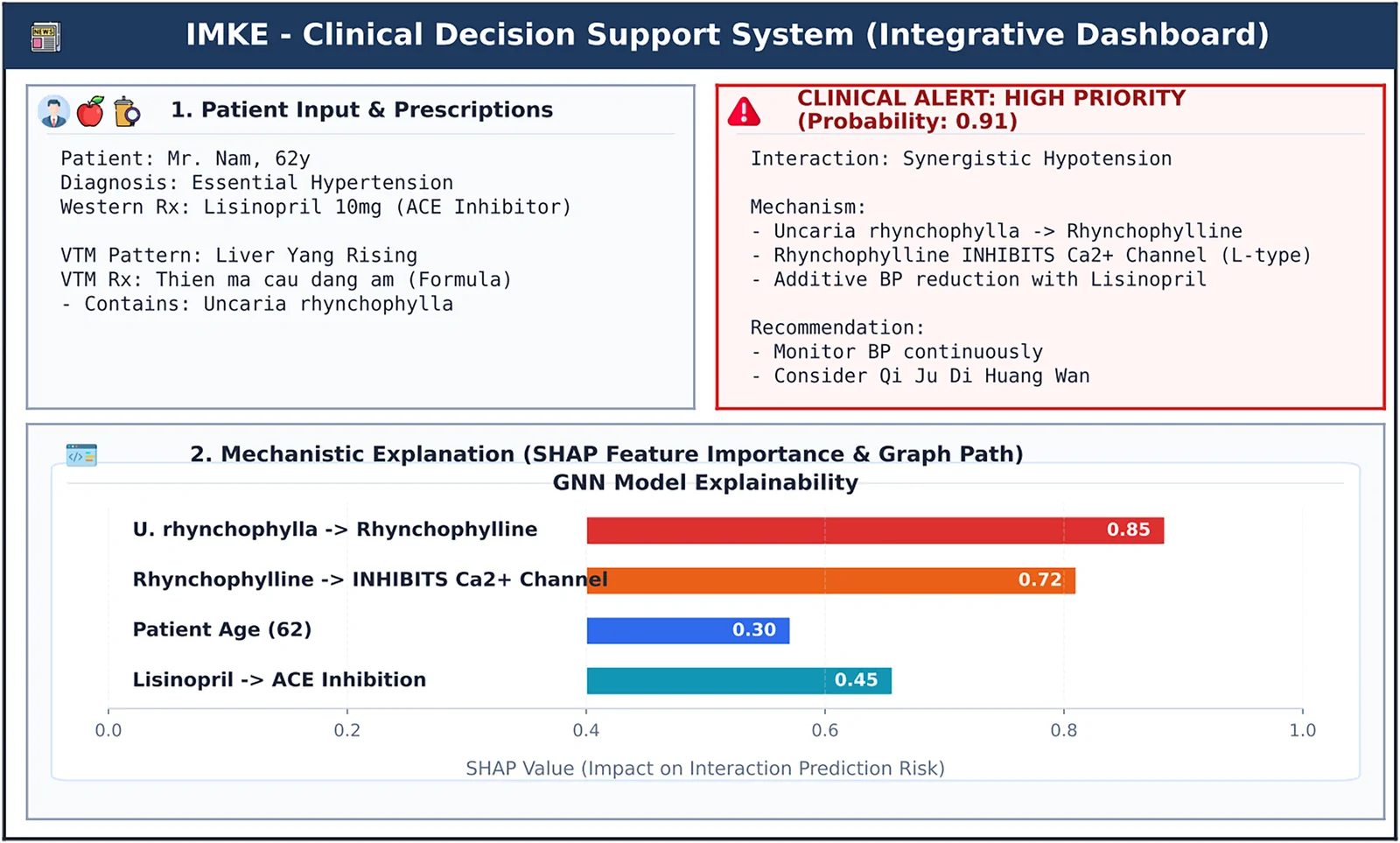
Simulated UI mockup of the IMKE Clinical Decision Support System dashboard, featuring dual-channel alerts and an illustrative attribution-plus-path explanation for a high-priority Herb-Drug Interaction. Numerical values and named explanation methods shown in the interface are examples used only to demonstrate the UI layout and do not represent empirical outputs or prescribed implementation choices.

#### 3.3.3 Explainability and integration pathways

To prevent alert fatigue and build clinician trust [59, 60], every IMKE alert should be accompanied by a reviewable computational rationale. Post-hoc feature-attribution frameworks, such as SHAP [61, 62], represent one illustrative option that could be applied to learned graph representations alongside explicit path tracing (e.g., identifying a candidate CYP450 inhibition pathway). Other explanation methods, including counterfactual, rule-based, and inherently interpretable graph approaches, remain eligible for comparison. For eventual deployment, the IMKE is envisioned to integrate with existing EHRs through interoperability standards such as HL7 FHIR APIs [63]; containerized microservices (e.g., Docker) are one possible deployment pattern for reducing healthcare IT integration friction [64].

It must be explicitly noted that attribution scores—including those produced by SHAP or alternative methods—describe computational rationale but do not inherently establish biological or causal mechanisms. All generated pathways and explanations would require review by clinical pharmacologists before clinical acceptance.

## 4 Conceptual framework illustration through simulated scenarios

To demonstrate the IMKE framework’s architectural design, reasoning capacity, and clinical utility, we present three simulated conceptual case studies spanning distinct chronic disease domains. Because this paper proposes an architectural blueprint, these scenarios do not represent live clinical trials but serve as theoretical step-by-step executions to demonstrate the data flow pipeline—from raw text input to NLP processing, graph traversal, GNN inference, and explainable CDSS output.

Each case study is designed to demonstrate a different category of herb-drug interaction mechanism (pharmacodynamic, pharmacokinetic, and toxicological). Table 5 details the specific interaction mechanisms mapped across the three scenarios and provides a comparative summary of the CDSS pipeline outputs.

**Table 5:** Comparative summary of IMKE simulated executions.

Scenario focus	Western medicine	VTM herb/formula	Illustrative interaction path	Conceptual CDSS output
CS-1: Hypertension	Lisinopril	Uncaria rhynchophylla	Pharmacodynamic (Calcium channel blocking overlap)	High Alert: Additive hypotension; Suggest alternative
CS-2: Diabetes	Glibenclamide	Momordica charantia	Pharmacodynamic (Insulin secretagogue synergy)	Synergy Alert: Risk of hypoglycemia; Optimize drug dosage
CS-3: Rheumatoid Arthritis	Methotrexate	Tripterygium wilfordii	Toxicological (CYP3A4-dependent triptolide exposure and additive hepatotoxicity)	High-priority alert: Avoid co-administration pending specialist review

### 4.1 Scenario 1: essential hypertension (pharmacodynamic synergism)

#### 4.1.1 Clinical profile

“Mr. Nam,” a 62-year-old male, presents with chronic headaches and dizziness. Western diagnosis: Essential Hypertension (ICD-10: I10), BP 150/95 mmHg, prescribed Lisinopril 10 mg (ACE inhibitor, DrugBank DB00722). VTM presentation: dizziness (huyễn vựng), tinnitus (nhĩ minh), irritability; pulse diagnosis: wiry and rapid (huyền sác); tongue: red with yellow coating. VTM pattern: Liver Yang Rising (Can dương thượng cang)—an upward surge of physiological energy due to Yin deficiency [28]. From a pharmacological perspective, in vitro and in vivo studies summarized by Ndagijimana et al. confirm that rhynchophylline, a major alkaloid in *Uncaria rhynchophylla*, exhibits significant calcium-channel-blocking activity [82]. This provides empirical support for potential additive hypotension when co-administered with ACE inhibitors.

#### 4.1.2 IMKE step-by-step execution pipeline

Step 1 (Clinical Input): The physician inputs the patient’s EHR data (Lisinopril) and unstructured VTM clinical notes into the CDSS interface.

Step 2 (NLP Processing): A context-aware Vietnamese clinical-language encoder would identify the key entities: [Western_Drug: Lisinopril], [VTM_Symptom: huyễn vựng], and [VTM_Pattern: Can dương thượng cang]. PhoBERT is one illustrative candidate for this function rather than a prescribed component.

Step 3 (Graph Traversal & Ontology Mapping): The entity-linking function would map the VTM pattern to Essential Hypertension through evidence-qualified shared phenotypes. A standards-compatible graph traversal—illustratively expressible using Neo4j/Cypher—could retrieve the MoH 2020 official integrative protocol [9] and the formula “Thiên ma câu đằng ẩm” (Gastrodia and Uncaria Decoction) [65], including [Herb: Uncaria rhynchophylla].

Step 4 (Relation-Aware Inference): A heterogeneous graph-learning method could evaluate the retrieved herb against the prescribed drug and generate a candidate high-risk alert from the topological evidence surrounding the edge (Uncaria rhynchophylla, INTERACTS_WITH, Lisinopril). R-GCN represents one possible instantiation.

Step 5 (Explainability & CDSS Output): An explanation layer could combine explicit path tracing with a suitable attribution method (e.g., SHAP) to identify graph evidence contributing to the alert. An illustrative mechanistic path is: Uncaria rhynchophylla → [CONTAINS] → Rhynchophylline → [INHIBITS] → Calcium Channel (L-type) → [VASODILATION].

Illustrative System Output: In this conceptual scenario, the CDSS would display a high-priority additive-hypotension alert and present monitoring or substitution options for clinician review rather than issue an autonomous recommendation.

#### 4.1.3 CDSS output

CLINICAL ALERT (High Priority): Potential Synergistic Hypotension. Mechanism: Rhynchophylline (Uncaria rhynchophylla) exhibits calcium channel blocking activity. Concurrent use with Lisinopril (ACE inhibitor) may produce additive blood pressure reduction, elevating risk of symptomatic hypotension (dizziness, syncope). Recommendation: (a) Monitor BP continuously during initiation; (b) Consider dose reduction of herbal formula; (c) Alternative formula substitution: Qi Ju Di Huang Wan (without Uncaria) may be appropriate for milder patterns.

### 4.2 Scenario 2: type 2 diabetes (therapeutic synergy discovery)

#### 4.2.1 Clinical profile

“Mrs. Lan,” a 58-year-old female, five-year history of poorly controlled Type 2 Diabetes (ICD-10: E11). Fasting glucose 9.4 mmol/L, HbA1c 8.7%. Current medications: Metformin 1000 mg twice daily (DrugBank DB00331), Glibenclamide 5 mg once daily (DrugBank DB01016). VTM presentation: fatigue, polydipsia, recurrent UTIs; pulse: deep, thin, weak (tram, te, nhuoc); tongue: pale with thin white coating. VTM pattern: Tiêu khát (wasting and thirsting disorder; Chinese: 消渴), characterized by Kidney-Spleen Qi deficiency. Clinical and pharmacological evaluations indicate that charantin-containing preparations derived from *Momordica charantia* can promote pancreatic beta-cell activity and insulin secretion [66, 67]. This supports a plausible biological mechanism for synergistic hypoglycemia when combined with sulfonylureas such as Glibenclamide.

#### 4.2.2 IMKE step-by-step execution pipeline

Step 1 (Clinical Input): The physician inputs Western meds (Metformin, Glibenclamide) and VTM symptoms.

Step 2 (NLP Processing): A context-aware clinical-language encoder would extract [Western_Drug: Glibenclamide], [Western_Drug: Metformin], and [VTM_Pattern: Tiêu khát].

Step 3 (Graph Traversal & Ontology Mapping): The IKG bridges Tiêu khát to Type 2 Diabetes (confidence weight ψ=0.82). Traversal of the MoH 2026 guidelines [10] retrieves the base formula *Lục vị địa hoàng hoàn*, supplemented clinically with [Herb: Momordica charantia] (Bitter melon).

Step 4 (Relation-Aware Inference): A future heterogeneous graph-learning module could evaluate the herb–drug pairs and generate a candidate interaction alert between Momordica charantia and Glibenclamide.

Step 5 (Explainability & CDSS Output): An illustrative combination of path tracing and post-hoc attribution could highlight the following biological pathway: Momordica charantia → [CONTAINS] → Charantin → [ACTIVATES] → Pancreatic Beta Cells → [INCREASES] → Insulin Secretion → [OVERLAPS MECHANISM WITH] → Glibenclamide [66, 67].

Illustrative System Output: In this conceptual scenario, the CDSS would present a dual-channel output: a potential compounded-hypoglycemia warning and a hypothesis-generating synergy flag requiring specialist assessment before any medication adjustment.

### 4.3 Scenario 3: rheumatoid arthritis (pharmacokinetic toxicity)

#### 4.3.1 Clinical profile

“Ms. Hoa,” a 55-year-old female, eight-year history of Rheumatoid Arthritis (ICD-10: M05). CRP 24 mg/L (elevated), ESR 58 mm/hr. Current medication: Methotrexate 15 mg/week (DrugBank DB00563). VTM presentation: bilateral joint pain worsened by cold and damp weather; pulse: wiry and tight (huyền khẩn); tongue: pale with white greasy coating. VTM pattern: Wind-Cold-Damp Bi Syndrome (phong hàn thấp tý), a pattern of external pathogenic obstruction of the musculoskeletal channels. Pharmacokinetic studies identify CYP3A4 as the predominant enzyme responsible for triptolide metabolism and show that reduced CYP3A4 activity can increase triptolide exposure and liver injury [83]. Thus, co-administration with hepatotoxic agents such as Methotrexate raises concern for additive liver toxicity, although a CYP3A4-mediated reduction in Methotrexate clearance has not been established.

#### 4.3.2 IMKE step-by-step execution pipeline

Step 1 (Clinical Input): Input of Methotrexate prescription and VTM musculoskeletal symptoms.

Step 2 (NLP Processing): Extraction of [Western_Drug: Methotrexate] and [VTM_Pattern: Phong hàn thấp tý].

Step 3 (Graph Traversal & Ontology Mapping): The IKG conceptually maps the pattern to RA (ICD-10: M05) [9]. A graph query—with Cypher serving only as an illustrative syntax—could retrieve Quyên bí phương (Juan Bi Fang, 4), which may clinically include [Herb: Tripterygium wilfordii] (Thunder God Vine).

Step 4 (Relation-Aware Inference): A heterogeneous graph-learning method could flag a candidate toxicological risk based on shared metabolic pathways between the herb and the drug.

Step 5 (Explainability & CDSS Output): A candidate explanation method could highlight the following potential toxicological path: Tripterygium wilfordii [CONTAINS] Triptolide [METABOLIZED BY] Hepatic CYP3A4 [REDUCED CYP3A4 ACTIVITY] Increased Triptolide Exposure [ADDITIVE HEPATOTOXICITY WITH] Methotrexate [68, 83].

Illustrative System Output: In this conceptual scenario, the CDSS would present a high-priority hepatotoxicity concern, identify the supporting evidence path, and defer avoidance or substitution decisions to specialist review.

#### 4.3.3 CDSS output

CLINICAL ALERT (High Priority): Potential Additive Hepatotoxicity. Mechanism: CYP3A4 is a major metabolic pathway for triptolide; reduced CYP3A4 activity may increase triptolide exposure and compound the hepatic burden of Methotrexate. This pathway does not establish CYP3A4-mediated inhibition of Methotrexate clearance and therefore requires pharmacologist review. Recommendation: Avoid co-administration unless supported by specialist assessment and close hepatic monitoring. Substitute: Juan Bi Fang reformulated without Tripterygium wilfordii; consider *Du Huo Ji Sheng Wan* as an alternative Wind-Damp formula with a safer interaction profile.

### 4.4 Architectural synthesis across scenarios

These three simulated executions collectively illustrate four hypothesized properties of the IMKE architecture. First, Generalizability: the functional pipeline could be instantiated across distinct domains without disease-specific redesign. Second, Mechanism Diversity: relation-aware reasoning may represent synergistic, additive, and toxicological pathways. Third, Bidirectional Value: the framework is intended to support both risk identification and investigation of potential therapeutic synergies. Finally, Explainability: candidate outputs should be accompanied by traceable computational paths that clinicians can review, without treating those paths as proof of causality [59].

## 5 Perspective and discussion: open challenges and future roadmap

The IMKE framework represents a paradigm shift in the clinical operationalization of integrative medicine. By providing a structured, computable bridge between VTM and Western pharmacology, it transforms integrative practice from an informal art dependent on individual practitioner memory into a systematically supported, safety-conscious clinical discipline. However, transitioning this architectural blueprint into a globally deployable clinical reality requires navigating profound technical, linguistic, and regulatory barriers.

### 5.1 Clinical and institutional implications

Critically, the IMKE is not designed in a regulatory vacuum. It is explicitly grounded in Vietnam’s Ministry of Health official integrative medicine guidelines (2020, 2026). This alignment provides regulatory legitimacy, ensuring that CDSS recommendations carry the institutional authority necessary to overcome clinician alert fatigue. Furthermore, the MoH guidelines explicitly mandate the combination of Western diagnostic methods with traditional treatments. The IMKE operationalizes this mandate by formalizing cross-paradigm safety: the GNN-based HDI prediction module closes the shadow pharmacovigilance gap, converting a known clinical blind spot—where over 42% of patients concurrently use undisclosed traditional medicines—into a manageable, continuously monitored risk.

Beyond safety, the IMKE acts as an engine for cultural preservation. By digitizing unstructured classical texts and encoding them into a heterogeneous multigraph, it ensures the “art” of traditional healing is preserved in a durable, computable format while maintaining its ontological integrity—representing VTM patterns in their native conceptual vocabulary without arbitrarily collapsing them into Western disease categories.

### 5.2 Perspective: overcoming open challenges

To realize the full potential of the IMKE architecture, the research and medical communities must collaboratively address three formidable barriers.

#### 5.2.1 The data scarcity and cold-start bottleneck

Unlike Traditional Chinese Medicine (TCM), which benefits from large digitized resources (e.g., TCMID, HERB), VTM suffers from severe labeled-data scarcity. Any data-driven relation-learning approach for HDI prediction would require sufficiently representative positive and negative interaction evidence. Directly applying high-capacity models to sparse VTM data therefore risks a cold-start problem and unreliable representations.

Potential methodological pathways include cross-lingual transfer learning [69], ontology alignment where pharmacological overlap exists, and human-in-the-loop active learning [70]. Future studies could compare transferred representations, weak supervision, retrieval-augmented methods, and expert-curated baselines. Low-confidence predictions could be routed to clinical pharmacologists and VTM practitioners, but the value and safety of iterative model updating must be established empirically.

#### 5.2.2 Semantic ambiguity in classical texts

VTM texts present extreme linguistic complexity. A critical barrier for the NLP pipeline is semantic ambiguity, where a single classical term holds vastly different meanings depending on its context. For instance, the concept of “Khí” (Qi – 汽) can refer to literal air, vital physiological energy, a specific organ’s functional state (e.g., Spleen Qi), or even external pathogenic factors (e.g., Cold Qi).

Static word embeddings, such as Word2Vec [71] or GloVe [72], cannot represent context-dependent meanings with a single fixed vector. Deep contextualized representations [73] therefore provide a plausible methodological direction, potentially supplemented by knowledge-aware encoding [53]. A future implementation could couple a Vietnamese contextual encoder (with PhoBERT as one candidate) to graph-derived context, but whether this improves disambiguation over multilingual, retrieval-based, or rule-assisted alternatives remains an empirical question.

#### 5.2.3 Regulatory frameworks and liability imperatives

Perhaps the most daunting challenge is not computational, but legal: If the IMKE predicts a drug combination is safe, and a patient subsequently suffers fatal hepatotoxicity, who bears the liability? The software developer, the hospital, or the prescribing physician? As Lipton notes regarding the “mythos of model interpretability” [74], and Holzinger et al. argue, the boundary between “decision support” and “clinical authority” becomes dangerously blurred when clinicians lack the expertise to critically evaluate AI outputs [59].

Any future IMKE implementation would need to be evaluated against applicable medical-device and AI governance frameworks [75, 76]. The framework is conceived as clinician-facing decision support rather than an autonomous prescriber, with final clinical authority remaining with qualified practitioners. This governance requirement motivates a method-agnostic explanation layer: attribution, path tracing, counterfactuals, rule-based rationale, and inherently interpretable approaches should be compared for their ability to support independent clinical review.

### 5.3 Ethical governance and cultural inclusion

The deployment of AI in integrative healthcare introduces profound ethical responsibilities. The medicinal practices of Vietnam’s 54 recognized ethnic minority groups (e.g., the Tay, Nung, H’Mong) are severely underrepresented in existing digitized academic archives. Without deliberate corpus curation strategies targeting these communities, the IMKE’s IKG risks algorithmically marginalizing minority therapeutic traditions, inadvertently entrenching existing health inequities. Mitigation requires targeted digitization partnerships with regional hospitals and community health workers. Furthermore, full clinical utility depends on integrating sensitive EHR and VTM consultation data. Strict adherence to data governance obligations, including localized federated learning techniques to preserve patient privacy [77], is essential. Furthermore, aligning the IMKE with Vietnam’s ongoing digital health transformation [78] requires parallel investments in digital literacy to ensure the health workforce is fully equipped to utilize AI-augmented systems [79].

### 5.4 Strategic roadmap to clinical reality

Transitioning the IMKE from a conceptual architectural blueprint to clinical evaluation can be organized as a non-prescriptive three-phase research pathway (illustrated in Figure 7).

**Figure 7:**
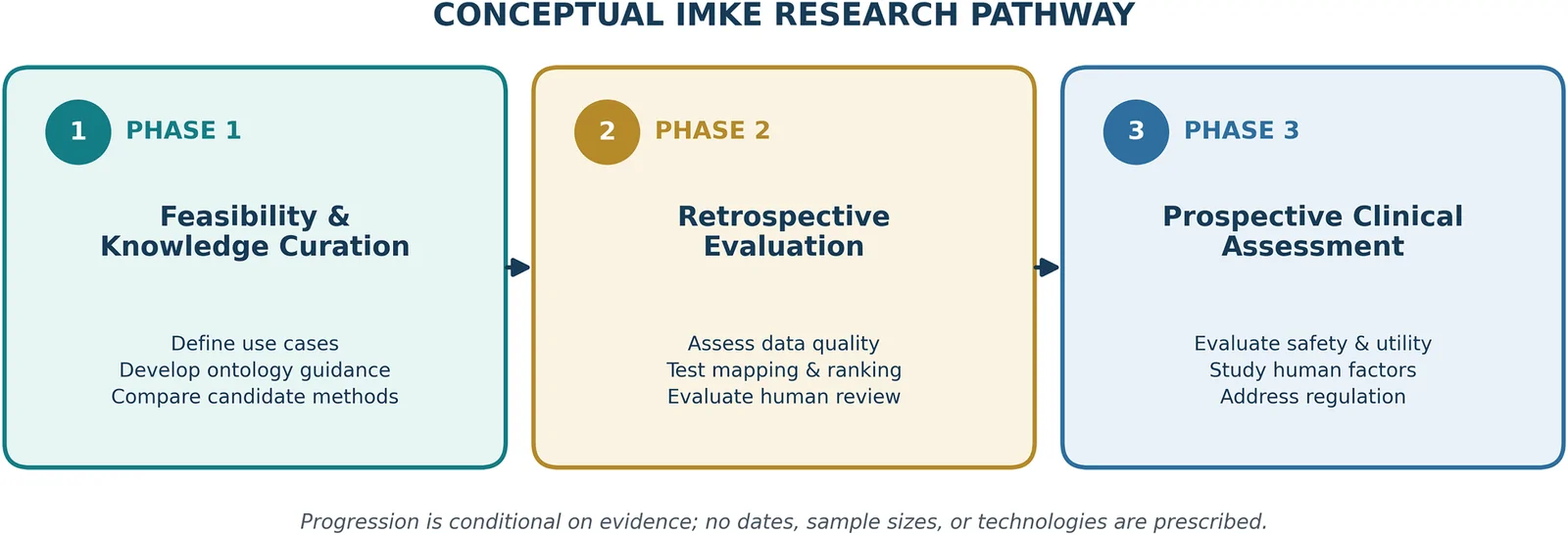
Conceptual IMKE research pathway. The sequence illustrates candidate phases from feasibility design to retrospective evaluation and prospective clinical assessment; it does not prescribe dates, sample sizes, or implementation technologies.

Phase 1—Feasibility and Knowledge Curation: Define representative clinical use cases, develop ontology and annotation guidance, and compare alternative language-processing, graph-storage, and reasoning approaches on a pilot corpus.

Phase 2—Retrospective Evaluation: Assess data quality, mapping reliability, interaction-ranking performance, explanation usefulness, and workflow feasibility using appropriately governed retrospective records and expert review.

Phase 3—Prospective Clinical Assessment: If feasibility and retrospective evidence are adequate, evaluate safety, clinical utility, human factors, and regulatory requirements in prospectively designed studies before considering adaptation to TCM, Ayurveda [80], Kampo [81], or other traditional medicine systems.

Table 6 outlines conceptual evaluation dimensions that future protocols could operationalize through prospectively defined metrics and acceptance criteria.

**Table 6:** Conceptual evaluation dimensions for future empirical studies of IMKE.

Functional requirement	Candidate evaluation dimensions	Criteria to be pre-specified	Illustrative validation approach
Clinical-language concept extraction	Precision, recall, F1-score, robustness across linguistic registers	Baselines, subgroup analyses, and acceptable error profiles defined before testing	Annotated VTM corpus with comparison across multilingual, Vietnamese-specific, domain-adapted, and rule-assisted methods
Ontology alignment and knowledge integration	Mapping accuracy, coverage, semantic consistency, provenance quality	Clinically acceptable mapping and evidence-quality criteria established prospectively	Blinded expert review of sampled VTM–biomedical mappings and ontology consistency checks
Relation-aware interaction ranking	Discrimination, calibration, ranking quality, generalization to unseen entities	Task-specific acceptance criteria and comparator methods registered before evaluation	Held-out HDI links, temporal validation, external knowledge bases, and ablation studies across candidate graph methods
Clinical decision support	Alert precision, recall, acceptance, override patterns, alert burden, safety outcomes	Clinician-centered utility and safety criteria co-designed before deployment	Simulation studies followed by staged usability and prospective clinical evaluation if justified
System interoperability and performance	Response time, reliability, scalability, standards compliance	Workflow-compatible service levels defined for the intended deployment setting	Testing under simulated and controlled clinical workloads using alternative deployment architectures
Explanation and human review	Fidelity, stability, path plausibility, counterfactual usefulness, user comprehension	Explanation-quality criteria established with clinicians and pharmacologists	Comparative evaluation of attribution, path-based, counterfactual, rule-based, and inherently interpretable methods

This table specifies evaluation dimensions rather than achieved results, fixed numerical targets, or a finalized protocol. Metrics, comparators, thresholds, datasets, and candidate technologies must be prospectively justified in future empirical studies.

## 6 Conclusion

This paper has presented a comprehensive review of Artificial Intelligence in traditional medicine and introduced the Integrative Medicine Knowledge Engine (IMKE) as a novel architectural perspective. By formally addressing the three foundational barriers to safe clinical integration—the Knowledge Gap, the Evidence Gap, and the Safety Gap—the IMKE framework provides a technically rigorous, mathematically grounded, and culturally sensitive blueprint for 21st-century integrative healthcare.

The proposed three-stage functional pathway comprises context-aware clinical-language processing, a heterogeneous Integrative Knowledge Graph (IKG), and relation-aware safety reasoning delivered through an explainable clinical interface. PhoBERT, Neo4j, R-GCN, SHAP, and related technologies are examples of possible instantiations rather than required components. Through simulated scenarios spanning three chronic disease domains, this paper illustrated hypotheses about how the architecture could organize cross-paradigm herb–drug interaction evidence and potential therapeutic synergies; it did not evaluate a trained model or establish clinical performance.

Beyond its immediate technical contributions, the IMKE architecture establishes a computational model for cultural preservation. By algorithmically encoding VTM’s rich therapeutic heritage into a durable, multi-relational knowledge structure, the framework ensures that centuries of phenomenological medical wisdom are preserved with ontological integrity and systematically evolved into a safer, evidence-informed clinical tradition. Furthermore, its agnostic, modular design provides a globally scalable template that can be readily adapted to harmonize other traditional medical systems—from TCM and Ayurveda [80] to Kampo [81] and Unani medicine—with modern Western biomedicine.

Transitioning this conceptual blueprint into clinical reality will require sustained, interdisciplinary collaboration spanning computer science, clinical pharmacology, bioethics, and healthcare policy. As detailed in our perspective, navigating the formidable bottlenecks of VTM data scarcity, linguistic ambiguity, and stringent regulatory liabilities will define the next phase of empirical research. Ultimately, as Artificial Intelligence continues to disrupt global healthcare, the IMKE framework demonstrates that the most profound technological opportunities lie not in replacing human clinical intuition, but in building the interoperable computational infrastructure required to connect, validate, and amplify the full breadth of humanity’s healing knowledge.

## Data availability statement

The original contributions presented in the study are included in the article/Supplementary Material, further inquiries can be directed to the corresponding author.

## Footnotes

^1^https://www.yhct.vn/.

## References

[1] World Health Organization. World health statistics 2024: monitoring health for the SDGs, sustainable development goals, Who.int. World Health Organization, 2024. [Online].

[2] Wang S Li X. A hypothesis of cybernetic medicine: redefining health and disease through control theory. Med Hypotheses. (2025) 205:111823. 10.1016/j.mehy.2025.111823

[3] Khan SN Danishuddin Khan MWA Guarnera L Akhtar SMF. Multi-modal AI in precision medicine: integrating genomics, imaging, and EHR data for clinical insights. Front Artif Intell. (2025) 8:1743921. 10.3389/frai.2025.1743921 41573270 PMC12819606

[4] Zhou M-C Fei Y-T Lai X-Z Lan J. Liu B. Wang Z-W, et al. Progress and challenges in integrated traditional Chinese and western medicine in China from 2002 to 2021. Front Pharmacol. (2024) 15:1425940. 10.3389/fphar.2024.1425940 39309004 PMC11412861

[5] Béres A. Revisiting the relationship between traditional East Asian medicine and biomedicine: incorporating the Western into the Eastern. Eur J Integr Med. (2024) 70:102388. 10.1016/j.eujim.2024.102388

[6] Van Sam H Baas P Keßler PJA. Traditional medicinal plants in Ben En National Park, Vietnam. Blumea. (2008) 53(3):569–601. 10.3767/000651908X607521

[7] Hua OH Tran QTT Trinh D-TT Nguyen V-D Duong DPN Nguyen TT. A review of traditional uses, phytochemistry and pharmacological properties of some Vietnamese wound-healing medicinal plants. Nat Prod Commun. (2022) 17(4):1934578X2210883. 10.1177/1934578X221088379

[8] World Health Organization. Global traditional medicine strategy 2025-2034. Who.int (2025). Available online at: https://www.who.int/publications/i/item/9789240113176 (Accessed April 2, 2026).

[9] Bộ Y tế. Quyết định số 5013/QĐ-BYT ngày 01 tháng 12 năm 2020 về việc ban hành tài liệu chuyên môn: Hướng dẫn chẩn đoán và điều trị bệnh theo y học cổ truyền, kết hợp y học cổ truyền với y học hiện đại (Tập I) Hà Nội: Bộ Y tế (2020).

[10] Wu D Liu W Wang J Chen W Shi M Zhang L, et al. Clinical application guideline of combination with traditional Chinese medicine and Western Medicine in the prevention and treatment of chronic Obstructive Pulmonary Disease (2024). J Evid Based Med. (2025) 18(2):e70024. 10.1111/jebm.70024 40302135 PMC12041629

[11] Lu L Lu T Tian C Zhang X. AI: bridging ancient wisdom and modern innovation in traditional Chinese medicine. JMIR Med Inform. (2024) 12:e58491. 10.2196/58491 38941141 PMC11245652

[12] Nguyen DQ Tuan Nguyen A. PhoBERT: pre-trained language models for Vietnamese. In: *Findings of the association for computational linguistics: EMNLP 2020* (Stroudsburg, PA, USA: Association for Computational Linguistics), 2020.

[13] Adorisio S Fierabracci A Rossetto A Muscari I Nardicchi V Liberati AM, et al. Integration of traditional and Western medicine in Vietnamese populations: a review of health perceptions and therapies. Nat Prod Commun. (2016) 11(9):1409–16. 10.1177/1934578X1601100949 30807048

[14] Monnais L Tousignant N. The colonial life of pharmaceuticals: accessibility to healthcare, consumption of medicines, and medical pluralism in french Vietnam, 1905–1945. J Vietnam Stud. (2006) 1(1–2):131–66. 10.1525/vs.2006.1.1-2.131

[15] Sanghvi K Subraya CK Pai V Holavanna AR Nanjundaiah H Kunhikatta V. Pharmacovigilance in herbal drugs: a challenge. Curr Drug Saf. (2023) 18(2):138–42. 10.2174/1574886317666220428125943 36883268

[16] Li M Wang Y Chen Y Dong L Liu J Dong Y, et al. A comprehensive review on pharmacokinetic mechanism of herb-herb/drug interactions in Chinese herbal formula. Pharmacol Ther. (2024) 264:108728. 10.1016/j.pharmthera.2024.108728 39389315

[17] Thirunavukarasu AJ Ting DSJ Elangovan K Gutierrez L Tan TF Ting DSW. Large language models in medicine. Nat Med. (2023) 29(8):1930–40. 10.1038/s41591-023-02448-8 37460753

[18] Chandak P Huang K Zitnik M. Building a knowledge graph to enable precision medicine. Sci Data. (2023) 10(1):67. 10.1038/s41597-023-01960-3 36732524 PMC9893183

[19] Zhao X Wang Y Li P Xu J Sun Y Qiu M, et al. The construction of a TCM knowledge graph and application of potential knowledge discovery in diabetic kidney disease by integrating diagnosis and treatment guidelines and real-world clinical data. Front Pharmacol. (2023) 14:1147677. 10.3389/fphar.2023.1147677 37324451 PMC10264574

[20] Nisar U Ashraf H Jhanjhi NZ Ashfaq F Ihsan U Lattu A. Graph neural networks for drug–drug interaction prediction—predicting safe drug pairings with AI. In: *The 7th international global conference series on ICT integration in technical education & smart society* (Basel Switzerland: MDPI) (2025), 42.

[21] Miotto R Wang F Wang S Jiang X Dudley JT. Deep learning for healthcare: review, opportunities and challenges. Brief Bioinform. (2018) 19(6):1236–46. 10.1093/bib/bbx044 28481991 PMC6455466

[22] Obermeyer Z Emanuel EJ. Predicting the future-big data, machine learning, and clinical medicine. N Engl J Med. (2016) 375(13):1216–9. 10.1056/NEJMp1606181 27682033 PMC5070532

[23] Xue J Xu Z Wang Q Hou H Wei L Zhang J, et al. Clinical practice guidelines for prevention and treatment of postoperative gastrointestinal disorder with Integrated Traditional Chinese and Western Medicine (2023). J Evid Based Med. (2024) 17(1):207–23. 10.1111/jebm.12587 38530771

[24] Fang S Dong L Liu L Guo J Zhao L Zhang J, et al. HERB: a high-throughput experiment- and reference-guided database of traditional Chinese medicine. Nucleic Acids Res. (2021) 49(D1):D1197–206. 10.1093/nar/gkaa1063 33264402 PMC7779036

[25] Wu Y Zhang F Yang K Fang S Bu D Li H, et al. SymMap: an integrative database of traditional Chinese medicine enhanced by symptom mapping. Nucleic Acids Res. (2019) 47(D1):D1110–7. 10.1093/nar/gky1021 30380087 PMC6323958

[26] Qu X Tian Z Cui J Li R Li D Zhang X. A review of knowledge graph in traditional Chinese medicine: analysis, construction, application and prospects. Comput Mater Contin. (2024) 81(3):3583–616. 10.32604/cmc.2024.055671

[27] Sutton RT Pincock D Baumgart DC Sadowski DC Fedorak RN Kroeker KI. An overview of clinical decision support systems: benefits, risks, and strategies for success. NPJ Digit Med. (2020) 3(1):17. 10.1038/s41746-020-0221-y 32047862 PMC7005290

[28] Rakel DP Minichiello V, editors. Integrative medicine. 5th ed. Philadelphia, PA: Elsevier-Health Sciences Division (2022).

[29] Topol EJ. High-performance medicine: the convergence of human and artificial intelligence. Nat Med. (2019) 25(1):44–56. 10.1038/s41591-018-0300-7 30617339

[30] Li S Zhang B. Traditional Chinese medicine network pharmacology: theory, methodology and application. Chin J Nat Med. (2013) 11(2):110–20. 10.3724/SP.J.1009.2013.00110 23787177

[31] Doğan RI Leaman R Lu Z. NCBI disease corpus: a resource for disease name recognition and concept normalization. J Biomed Inform. (2014) 47:1–10. 10.1016/j.jbi.2013.12.006 PMC3951655 24393765

[32] Vaswani A Shazeer N Parmar N Uszkoreit J Jones L Gomez AN, et al. Attention is all you need. *arXiv* [Preprint]. *arXiv [cs.CL]* (2017).

[33] Lee J Yoon W Kim S Kim D Kim S So CH, et al. BioBERT: a pre-trained biomedical language representation model for biomedical text mining. Bioinformatics. (2020) 36(4):1234–40. 10.1093/bioinformatics/btz682 31501885 PMC7703786

[34] Alsentzer E Murphy J Boag W Weng W-H Jindi D Naumann T. Publicly available clinical BERT embeddings. In: Rumshisky A Roberts K Bethard S Naumann T, editors. Proceedings of the 2nd Clinical Natural Language Processing Workshop. Stroudsburg, PA, USA: Association for Computational Linguistics (2019). p. 72–8.

[35] Beltagy I Lo K Cohan A. SciBERT: a pretrained language model for scientific text. In: *Proceedings of the 2019 conference on empirical methods in natural language processing and the 9th international joint conference on natural language processing (EMNLP-IJCNLP)* (Stroudsburg, PA, USA: Association for Computational Linguistics) (2019).

[36] Hogan A Blomqvist E Cochez M d’Amato C de Melo G Gutiérrez C, et al. Knowledge graphs. ACM Comput Surv. (2022) 54(4):1–37. 10.1145/3447772

[37] Ji S Pan S Cambria E Marttinen P Yu PS. A survey on knowledge graphs: representation, acquisition, and applications. IEEE Trans Neural Netw Learn Syst. (2022) 33(2):494–514. 10.1109/TNNLS.2021.3070843 33900922

[38] Schlichtkrull M Kipf TN Bloem P van den Berg R Titov I Welling M. Modeling relational data with graph convolutional networks. In: Gangemi A Navigli R Vidal M-E Hitzler P Troncy R Hollink L, editors. The SemanticWeb. Cham: Springer International Publishing (2018). p. 593–607.

[39] Wang C Wang C Wang R, Research on the construction and application of knowledge graph of traditional Chinese medicine formulas. In: Proceedings of the 2024 International Conference on Biomedicine and Intelligent Technology. New York, NY, USA: ACM (2024). p. 26–33.

[40] Zhang B Li R Yin K Hua S Li S Jiang M, et al. Construction and application of traditional Chinese Medicine knowledge graph based on Large Language Model. Interdiscip Sci. (2026) 18(2):734–46. 10.1007/s12539-025-00735-1 40603824

[41] Zhu W Zhang Y She K Chen J. Artificial intelligence-driven intelligentization of traditional Chinese medicine diagnosis: applications, challenges, and prospects of multimodal fusion and large language model. J Tradit Chin Med Sci. (2026) 13(3):291–301. 10.1016/j.jtcms.2026.05.002

[42] Hopkins AL. Network pharmacology: the next paradigm in drug discovery. Nat Chem Biol. (2008) 4(11):682–90. 10.1038/nchembio.118 18936753

[43] Huang L Xie D Yu Y Liu H Shi Y Shi T, et al. TCMID 2.0: a comprehensive resource for TCM. Nucleic Acids Res. (2018) 46(D1):D1117–20. 10.1093/nar/gkx1028 29106634 PMC5753259

[44] Chen KC Lu R Iqbal U Hsu K-C Chen B-L Nguyen P-A, et al. Interactions between traditional Chinese medicine and western drugs in Taiwan: a population-based study. Comput Methods Programs Biomed. (2015) 122(3):462–70. 10.1016/j.cmpb.2015.09.006 26470816

[45] Tatonetti NP Ye PP Daneshjou R Altman RB. Data-driven prediction of drug effects and interactions. Sci Transl Med. (2012) 4(125):125ra31. 10.1126/scitranslmed.3003377 22422992 PMC3382018

[46] Tamir A Yuan J-S. KITE-DDI: a knowledge graph integrated transformer model for accurately predicting drug-drug interaction events from drug SMILES and biomedical knowledge graph. IEEE Access. (2025) 13:40028–43. 10.1109/ACCESS.2025.3547594

[47] Kundi IN Sheikh SA Malik FM Bhatti KA. DDI-KGAT: a graph attention network on biomedical knowledge graph for the prediction of drug-drug interactions. IEEE Access. (2024) 12:162028–39. 10.1109/ACCESS.2024.3483993

[48] Vilar S Friedman C Hripcsak G. Detection of drug-drug interactions through data mining studies using clinical sources, scientific literature and social media. Brief Bioinform. (2018) 19(5):863–77. 10.1093/bib/bbx010 28334070 PMC6454455

[49] Spanakis M Tzamali E Tzedakis G Koumpouzi C Pediaditis M Tsatsakis A, et al. Artificial intelligence models and tools for the assessment of drug–herb interactions. Pharmaceuticals. (2025) 18(3):282. 10.3390/ph18030282 40143062 PMC11944892

[50] Paul SG Saha A Hasan MZ Noori SRH Moustafa A. A systematic review of graph neural network in healthcare-based applications: recent advances, trends, and future directions. IEEE Access. (2024) 12:15145–70. 10.1109/ACCESS.2024.3354809

[51] Ladinsky JL Volk ND Robinson M. The influence of traditional medicine in shaping medical care practices in Vietnam today. Soc Sci Med. (1987) 25(10):1105–10. 10.1016/0277-9536(87)90351-0 3317871

[52] Nguyen AT Ngo KH Ngo NX Le NA Trinh Phuong N Thao DT, et al. Value chain analysis of medicinal plants in Geoparks: livelihoods of ethnic minorities based on non-timber forest products (NTFPs) in Vietnam. PLoS One. (2025) 20(7):e0324746. 10.1371/journal.pone.0324746 40700426 PMC12286371

[53] Wei C-H Peng Y Leaman R Davis AP Mattingly CJ Li J, et al. Assessing the state of the art in biomedical relation extraction: overview of the BioCreative V chemical-disease relation (CDR) task. Database (Oxford). (2016) 2016:baw032. 10.1093/database/baw032 26994911 PMC4799720

[54] Zhang M Moalin M Haenen GRMM. Connecting west and east. Int J Mol Sci. (2019) 20(9):2333. 10.3390/ijms20092333 31083489 PMC6540579

[55] Wishart DS Feunang YD Guo AC Lo EJ Marcu A Grant JR, et al. DrugBank 5.0: a major update to the DrugBank database for 2018. Nucleic Acids Res. (2018) 46(D1):D1074–82. 10.1093/nar/gkx1037 29126136 PMC5753335

[56] Kipf TN Welling M. Semi-supervised classification with graph convolutional networks. *arXiv* [Preprint]. *arXiv [cs.LG]* (2016).

[57] Veličković P Cucurull G Casanova A Romero A Li P Bengio Y. Graph Attention Networks, arXiv [stat.ML]. (2017).

[58] Hamilton WL Ying R Leskovec J. Inductive representation learning on large graphs. *arXiv* [Preprint]. *arXiv [cs.SI]* (2017).

[59] Holzinger A Biemann C Pattichis CS Kell DB. What do we need to build explainable AI systems for the medical domain?, arXiv [cs.AI]. (2017).

[60] Shortliffe EH Sepúlveda MJ. Clinical decision support in the era of artificial intelligence. JAMA. (2018) 320(21):2199–200. 10.1001/jama.2018.17163 30398550

[61] Lundberg S Lee S-I. A unified approach to interpreting model predictions. *arXiv* [Preprint]. *arXiv [cs.AI]* (2017).

[62] Doshi-Velez F Kim B. Towards a rigorous science of interpretable machine learning. *arXiv* [Preprint]. *arXiv [stat.ML]* (2017).

[63] Lehne M Sass J Essenwanger A Schepers J Thun S. Why digital medicine depends on interoperability. NPJ Digit Med. (2019) 2(1):79. 10.1038/s41746-019-0158-1 31453374 PMC6702215

[64] Sittig DF Singh H. A new sociotechnical model for studying health information technology in complex adaptive healthcare systems. Qual Saf Health Care. (2010) 19(Suppl. 3):i68–i74. 10.1136/qshc.2010.042085 20959322 PMC3120130

[65] Chen B Wang Y He Z Wang D Yan X Xie P. Tianma Gouteng decoction for essential hypertension: protocol for a systematic review and meta-analysis. Medicine (Baltimore). (2018) 97(8):e9972. 10.1097/MD.0000000000009972 29465596 PMC5841972

[66] Joseph B Jini D. Antidiabetic effects of *Momordica charantia* (bitter melon) and its medicinal potency. Asian Pac J Trop Dis. (2013) 3(2):93–102. 10.1016/S2222-1808(13)60052-3

[67] Fuangchan A Sonthisombat P Seubnukarn T Chanouan R Chotchaisuwat P Sirigulsatien V, et al. Hypoglycemic effect of bitter melon compared with metformin in newly diagnosed type 2 diabetes patients. J Ethnopharmacol. (2011) 134(2):422–8. 10.1016/j.jep.2010.12.045 21211558

[68] Shan Y Zhao J Wei K Jiang P Xu L Chang C, et al. A comprehensive review of Tripterygium wilfordii hook. f. in the treatment of rheumatic and autoimmune diseases: bioactive compounds, mechanisms of action, and future directions. Front Pharmacol. (2023) 14:1282610. 10.3389/fphar.2023.1282610 38027004 PMC10646552

[69] Pan SJ Yang Q. A survey on transfer learning. IEEE Trans Knowl Data Eng. (2010) 22(10):1345–59. 10.1109/TKDE.2009.191

[70] Steffny L Dahlem N Reichl L Gisa K Greff T Werth D, Design of a human-in-the-loop centered AI-based clinical decision support system for professional care planning. In: Lukowicz P Mayer S Koch J Shawe-Taylor J Tiddi I, editors. Frontiers in Artificial Intelligence and Applications. 2nd International Conference on Hybrid Human-Artificial Intelligence, held from 26-30 June 2023. Amsterdam: IOS Press (2023). p. 263–73. Available online at: https://ebooks.iospress.nl/volume/hhai-2023-augmenting-human-intellect-proceedings-of-the-second-international-conference-on-hybrid-human-artificial-intelligence

[71] Mikolov T Sutskever I Chen K Corrado G Dean J. Distributed Representations of Words and Phrases and their Compositionality, arXiv [cs.CL]. (2013).

[72] Pennington J Socher R Manning C. Glove: global vectors for word representation. In: *Proceedings of the 2014 conference on empirical methods in natural language processing (EMNLP)* (Stroudsburg, PA, USA: Association for Computational Linguistics) (2014).

[73] Peters ME Neumann M Iyyer M Gardner M Clark C Lee K. Deep contextualized word representations. In: Walker M Ji H Stent A, editors. Proceedings of the 2018 Conference of the North American Chapter of the Association for Computational Linguistics: Human Language Technologies, Volume 1 (Long Papers), Stroudsburg, PA, USA: Association for Computational Linguistics (2018). p. 2227–37.

[74] Lipton ZC. The mythos of model interpretability. Commun ACM. (2018) 61(10):36–43. 10.1145/3233231

[75] Yang S-R Chien J-T Lee C-Y. Advancements in clinical evaluation and regulatory frameworks for AI-driven software as a medical device (SaMD). IEEE Open J Eng Med Biol. (2025) 6:147–51. 10.1109/OJEMB.2024.3485534 39698124 PMC11655112

[76] Mesarčík M Solarova S Podroužek J Bieliková M. Stance on the proposal for a regulation laying down harmonised rules on Artificial Intelligence – Artificial Intelligence Act. *SocArXiv* [Preprint] (2022). 10.1007/s43681-022-00194-0 PMC9274635 35846557

[77] Rieke N Hancox J Li W Milletarì F Roth HR Albarqouni S, et al. The future of digital health with federated learning. npj Digit Med. (2020) 3(1):119. 10.1038/s41746-020-00323-1 33015372 PMC7490367

[78] Nguyen TA Nguyen TB Nguyen DC Vu AD Dang KL Le NQ, et al. What are the opportunities and challenges of using AI in medical education in Vietnam? JMIR Med Educ. (2025) 11:e77817. 10.2196/77817 41329943 PMC12671901

[79] McIsaac M Buchan J Abu-Agla A Kawar R Campbell J. Global strategy on human resources for health: workforce 2030-a five-year check-in. Hum Resour Health. (2024) 22(1):68. 10.1186/s12960-024-00940-x 39363378 PMC11450981

[80] Shaumya S Kumar R. Artificial intelligence in Ayurveda: A systematic review (2020-2025). J Ayurveda Naturopathy. (2025) 2(2):05–19. 10.33545/ayurveda.2025.v2.i2.A.24

[81] Watanabe K Matsuura K Gao P Hottenbacher L Tokunaga H Nishimura K, et al. Traditional Japanese Kampo medicine: clinical research between modernity and traditional medicine-the state of research and methodological suggestions for the future. Evid Based Complement Alternat Med. (2011) 2011:513842. 10.1093/ecam/neq067 21687585 PMC3114407

[82] Ndagijimana A Wang X Pan G Zhang F Feng H Olaleye O. A review on indole alkaloids isolated from Uncaria rhynchophylla and their pharmacological studies. Fitoterapia. (2013) 86:35–47. 10.1016/j.fitote.2013.01.018 23376412

[83] Xiao X Zhang T Huang J Zhao Q Li F. Effect of CYP3A4 on liver injury induced by triptolide. Biomed Chromatogr. (2020) 34(8):e4864. 10.1002/bmc.4864 32330997

[84] Jin Y-H Wang Y-P Xie Y-L Tian G-H Zhang X-Y Shi N-N, et al. Research on the development methodology for clinical practice guidelines for organic integration of traditional Chinese and Western medicine. Mil Med Res. (2023,) 10(1):45. 10.1186/s40779-023-00481-9 PMC10523673 37752599

[85] Xie Y-l Han F Jin Y-h Ding Y-b Guo J Ji D-x, et al. Organic integration of traditional Chinese and Western medicines—future of clinical practice guidelines of integrated traditional Chinese and Western medicines. Chin J Integr Med. (2024) 30(4):359–65. 10.1007/s11655-023-3739-9 37528326

[86] Zhang P Zhang D Zhou W Wang L Wang B Zhang T, et al. Network pharmacology: towards the artificial intelligence-based precision traditional Chinese medicine. Brief Bioinform. (2024) 25(1):bbad518. 10.1093/bib/bbad518 PMC10777171 38197310

[87] Wei X Yang X Zhang X Zhao C Jin Y Shang H, et al. Toward complex-systems evidence for Chinese medicine: a four-phase evidence framework. J Evid Based Med. (2026) 19(1):e70111. 10.1111/jebm.70111. 41808319

