# Supplementary material for: Harmonizing Vietnamese traditional and western medicine: a digital health blueprint and the IMKE architectural framework
Source: Front Digit Health. 2026 Oct 1;8:1928965. doi: 10.3389/fdgth.2026.1928965 (PMC13668813; doi:10.3389/fdgth.2026.1928965)
Supplement: Supplementary file 1 [file Datasheet1.pdf]

Supplementary Material

ARTICLE INFORMATION

Article Title: Harmonizing Vietnamese Traditional and Western Medicine: A Systematic Review and Digital Health Blueprint for Safe Clinical Integration
Journal: Frontiers in Digital Health

1 LIST OF ABBREVIATIONS

AI: Artificial Intelligence

CDSS: Clinical Decision Support System

EHR: Electronic Health Record

GNN / R-GCN: Graph Neural Network / Relational Graph Convolutional Network

HDI / DDI: Herb-Drug Interaction / Drug-Drug Interaction

IKG: Integrative Knowledge Graph

NLP / NER / RE: Natural Language Processing / Named Entity Recognition / Relation Extraction

PRISMA: Preferred Reporting Items for Systematic Reviews and Meta-Analyses

TCM / VTM: Traditional Chinese Medicine / Vietnamese Traditional Medicine

UMLS: Unified Medical Language System

2 REGULATORY AND CLINICAL CONTEXT OF VIETNAMESE TRADITIONAL MEDICINE (VTM)

To provide international readers with the necessary context for the IMKE framework, this section outlines the historical, clinical, and regulatory landscape of Vietnamese Traditional Medicine (VTM) and its formal integration with Western biomedicine in Vietnam.

2.1 Historical and Epistemological Context of VTM

While VTM shares foundational philosophies with Traditional Chinese Medicine (TCM)—such as Yin/Yang theory and the meridian system—it is a distinct medical system heavily adapted to the tropical climate and endemic flora of Southeast Asia. The core philosophy of VTM was established by the 14th-century physician Tuệ Tĩnh [1] with the principle "Nam dược trị Nam nhân" (Vietnamese medicine for Vietnamese people), focusing on the use of local botanical resources. In the 18th century, the scholar Hải Thượng Lãn Ông systematized VTM in his seminal encyclopedia Hải Thượng Y Tông Tâm Lĩnh [2], which remains the clinical cornerstone for VTM practitioners today.

[1] https://pubmed.ncbi.nlm.nih.gov/11719941/

[2] https://yhoccotruyenqd.vn/kien-thuc-yhct/Tac-pham-kinh-dien/hai-thuong-y-tong-tam-linh-34.html

2.2 Institutional Integration and Ministry of Health (MoH) Regulations

Vietnam is one of the few countries globally where traditional medicine and Western biomedicine operate as a formally integrated, dual-paradigm system within the public healthcare network. The Ministry of Health (MoH) [3] of Vietnam actively mandates this integration through strict legal and clinical frameworks:

Decision No. 3890/QĐ-BYT[4] (2020) & Updates (2026): Guidelines for Diagnosing and Treating Diseases According to Traditional Medicine, Combining Traditional and Modern Medicine. This landmark compendium establishes standardized clinical pathways for high-prevalence chronic diseases. It explicitly provides a 1-to-1 mapping between VTM pattern-based diagnoses (e.g., Liver Yang Rising) and Western ICD-10 codes (e.g., Essential Hypertension). Furthermore, it legally mandates that VTM practitioners must utilize Western clinical diagnostics (e.g., blood tests, imaging) to ensure patient safety before prescribing traditional herbal formulas.

Circular No. 02/2024/TT-BYT [5]: Regulations on the combined practice of traditional and modern medicine in healthcare facilities. This recent circular provides the concrete legal foundation for cross-disciplinary collaboration. It explicitly authorizes the concurrent prescribing of pharmaceutical drugs and herbal remedies, and mandates that hospital directors bear the responsibility for monitoring the safety of these integrative treatments.

2.3 The Need for Digital Mediation

Despite these robust regulatory frameworks, the actual clinical execution relies heavily on the individual physician's memory and expertise. Because there has been no centralized, digitized warning system (CDSS) to cross-check the safety of combining MoH-approved herbal formulas with Western pharmaceuticals, the risk of Herb-Drug Interactions (HDIs) remains a critical blind spot. The IMKE framework proposed in this paper serves as the computational infrastructure required to digitally operationalize these MoH mandates, ensuring that dual-paradigm prescribing is both legally compliant and technologically safeguarded.

3 SUPPLEMENTARY TABLES

[3] https://moh.gov.vn/

[4] https://kcb.vn/phac-do/quyet-dinh-so-3809-qd-byt-ngay-27-thang-8-nam-2019-ve-viec-b.html

[5] https://vanban.chinhphu.vn/?pageid=27160&docid=209938

Table S1. Supplementary Table 1: Nomenclature and Glossary of VTM Terms

VTM Term (Vietnamese)	English Translation / Concept	Clinical Implication in IMKE Architecture
Bát cương (Eight-Principle)	The eight fundamental diagnostic principles (Yin/Yang, Interior/Exterior, Cold/Heat, Deficiency/Excess).	Used as structural edge attributes in the Knowledge Graph for pattern differentiation.
Cổ phương (Classical Formula)	Standardized herbal prescriptions passed down through centuries of historical texts.	Serves as the high-confidence [TREATS] root node in the IKG schema.
Can dương thượng cường	Liver Yang Rising (Uprising).	Mapped via shared phenotypes to Western Essential Hypertension.
Tiêu khát	Wasting and thirsting disorder.	Mapped via ontology alignment to Western Type 2 Diabetes.
Phong hàn thấp tý	Wind-Cold-Damp Bi Syndrome.	Mapped via inflammatory phenotypes to Western Rheumatoid Arthritis.
Khí (Qi)	Vital energy or functional state of an organ.	Represents a challenge in NLP semantic disambiguation requiring context-aware embeddings.

Table S2. Supplementary Table 2: Comparative Dimensions of Western and Eastern (VTM/TCM) Medicine Paradigms

Dimension	Western Medicine	Eastern Medicine (VTM/TCM)	Integrative Potential
Philosophical basis	Reductionism; isolates specific biological targets	Holism; treats body as interconnected system	Combine precision targeting with systemic balance
Diagnostic approach	Lab tests, imaging, biomarkers (ICD-10/DSM)	Pattern recognition: pulse, tongue, symptom clusters	Dual-layer diagnosis enhances specificity
Treatment modality	Pharmaceutical drugs, surgery, radiotherapy	Herbal formulas, acupuncture, dietary therapy	Synergistic therapy; reduced pharmaceutical burden
Evidence standard	RCTs, meta-analyses, systematic reviews	Historical texts, experiential practice, case series	AI can bridge evidence gaps via network analysis
Chronic disease mgmt	Effective for acute exacerbations; limited holistic view	Long-term wellness; addresses root causes	Combined protocols reduce recurrence rates
Data structure	Structured EHR, HL7 FHIR standards	Unstructured texts, oral traditions, Sino-Vietnamese	NLP + KG needed for unification
Herb/drug safety	Pharmacovigilance databases (FDA, EMA)	Underreported; limited formal interaction tracking	IMKE IKG provides cross-system safety layer
Cultural dimension	Universal evidence-based protocols	Culturally embedded; varies by region	AI must preserve cultural nuance in recommendations
